# Distinct specificities of the HEMK2 protein methyltransferase in methylation of glutamine and lysine residues

**DOI:** 10.1002/pro.4897

**Published:** 2024-01-23

**Authors:** Sara Weirich, Gizem T. Ulu, Thyagarajan T. Chandrasekaran, Jana Kehl, Jasmin Schmid, Franziska Dorscht, Margarita Kublanovsky, Dan Levy, Albert Jeltsch

**Affiliations:** ^1^ Institute of Biochemistry and Technical Biochemistry, Department of Biochemistry University of Stuttgart Stuttgart Germany; ^2^ The Shraga Segal Department of Microbiology, Immunology and Genetics Ben‐Gurion University of the Negev Be'er‐Sheva Israel; ^3^ The National Institute for Biotechnology in the Negev Ben‐Gurion University of the Negev Be'er‐Sheva Israel

**Keywords:** H4K12me1, HEMK2, histone methylation, KMT9, protein glutamine methylation, protein lysine methylation, SETD6

## Abstract

The HEMK2 protein methyltransferase has been described as glutamine methyltransferase catalyzing ERF1‐Q185me1 and lysine methyltransferase catalyzing H4K12me1. Methylation of two distinct target residues is unique for this class of enzymes. To understand the specific catalytic adaptations of HEMK2 allowing it to master this chemically challenging task, we conducted a detailed investigation of the substrate sequence specificities of HEMK2 for Q‐ and K‐methylation. Our data show that HEMK2 prefers methylation of Q over K at peptide and protein level. Moreover, the ERF1 sequence is strongly preferred as substrate over the H4K12 sequence. With peptide SPOT array methylation experiments, we show that Q‐methylation preferentially occurs in a G‐Q‐X_3_‐R context, while K‐methylation prefers S/T at the first position of the motif. Based on this, we identified novel HEMK2 K‐methylation peptide substrates with sequences taken from human proteins which are methylated with high activity. Since H4K12 methylation by HEMK2 was very low, other protein lysine methyltransferases were examined for their ability to methylate the H4K12 site. We show that SETD6 has a high H4K12me1 methylation activity (about 1000‐times stronger than HEMK2) and this enzyme is mainly responsible for H4K12me1 in DU145 prostate cancer cells.

## 1 INTRODUCTION

In eukaryotes, a variety of different epigenetic mechanisms exist like DNA methylation, and several types of histone post‐translational modifications (PTMs) such as acetylation, methylation, phosphorylation, and ubiquitination (Allis & Jenuwein, 2016). Epigenome modifications are added to the chromatin where they control DNA‐protein interactions and regulate gene expression and cellular development (Allis & Jenuwein, 2016; Zhao et al., 2021). Among all chromatin PTMs, methylation is the most extensively studied one and mainly occurs at lysine residues. Several lysine side chains in the core histones can be mono‐, di‐, or trimethylated by specific protein lysine methyltransferases (PKMTs), and depending on the site and degree of methylation, it can signal either activation or repression of gene expression (Husmann & Gozani, 2019; Jambhekar et al., 2019). Besides the methylation of lysine residues in histones, proteomic studies identified lysine methylation in a large number of non‐histone proteins, where the methylation is involved in the regulation of protein stability, activity, localization as well as protein–protein interactions (Cornett et al., 2019; Zhang et al., 2015). In addition, methylation at arginine, glutamine, glutamate, histidine, asparagine, aspartate, and cysteine has been identified, which is introduced by protein methyltransferases (P) with a corresponding target residue specificity (Clarke, 2013). Despite many examples documenting very important biological roles of protein methylation, and many well documented disease connections, the analysis of the substrate spectrum of PKMTs and particularly protein glutamine methyltransferases (PQMTs) is still incomplete.

HEMK2 (aka KMT9/N6AMT1) is the most well‐studied example of a PQMT. It methylates the glutamine in the GGQ loop of the eukaryotic ribosomal release factor ERF1 (Q171 in human ERF1, Q185 in murine ERF1) that is important for translational termination (Heurgue‐Hamard et al., 2002; Nakahigashi et al., 2002; Figaro et al., 2008). Structures of bacterial, yeast and mammalian orthologs of HEMK2 revealed that the enzyme is a member of the Rossman fold methyltransferases with a central 7‐stranded β‐sheet, surrounded by α‐helices on both sides (Schubert et al., 2003; Yang et al., 2004; Graille et al., 2005; Metzger et al., 2019; Gao et al., 2020). HEMK2 requires the interaction partner TRMT112 for complete methyltransferase activity and stability (Figaro et al., 2008; Graille et al., 2005). Extensive hydrogen bonds promote the interaction between HEMK2 and TRMT112, and in the complex TRMT112 hides the hydrophobic surface of HEMK2 and stabilizes it against degradation.

Over the past couple of years, additional target residues and substrate proteins methylated by HEMK2 have been identified. It was found that the enzyme prefers methylation of G‐Q‐X_3_‐R motifs (Kusevic et al., 2016) and based on this additional protein substrates, CHD5 and NUT, were also shown to be methylated by HEMK2 (Kusevic et al., 2016). Unexpectedly in 2019, it was discovered that HEMK2 also has a lysine methyltransferase activity on lysine 12 of histone H4, generating H4K12me1 (Metzger et al., 2019). This methylation was shown to be involved in androgen‐independent prostate tumor cell proliferation. Interestingly, although the chemical challenges for methylation of these two different target residues are very distinct, structural studies showed that the methylation reactions occur in the same active site (Graille et al., 2005; Metzger et al., 2019). However, lysine methylation was found to be promoted by alkaline pH, because of the requirement of lysine deprotonation prior to the methylation reaction (Woodcock et al., 2019).

Overall, these findings are very interesting as it is very unusual for a protein methyltransferase to be able to methylate side‐chains of different target amino acid residues, Q and K in case of HEMK2. To get more information on the specific catalytic adaptations of HEMK2 allowing it to master this chemically challenging task, in this study we conducted a detailed investigation of the substrate sequence specificities of HEMK2 on Q‐ and K‐methylation substrates, using ERF1 and H4K12 or the corresponding Q‐to‐K or K‐to‐Q mutants as test substrates in most experiments. Our data clearly show that HEMK2 prefers methylation of Q over K as methylation target at peptide and protein level and additionally the ERF1 sequence context is preferred over the H4K12 sequence. We show that lysine methylation preferentially occurs in an S/T‐K‐X_3_‐R context and identified novel HEMK2 lysine methylation substrate peptides derived from human proteins which are methylated with high activity. Since the methylation of H4K12 by HEMK2 was very low, other PKMTs were examined for their potential ability to methylate the H4K12 site. We show that SETD6, a well‐established protein lysine monomethyltransferase with relatively broad substrate profile (Levy et al., 2011; Vershinin et al., 2016, 2021; Feldman et al., 2019; Kublanovsky et al., 2023), has a strong H4K12 methylation activity, which is about 1000‐fold higher than that of HEMK2. Finally, we document that SETD6 indeed is mainly responsible for introducing H4K12me1 in DU145 human prostate cancer cells.

## 2 RESULTS

### 2.1 HEMK2 prefers methylation of Q over K at peptide level

**FIGURE 1:**
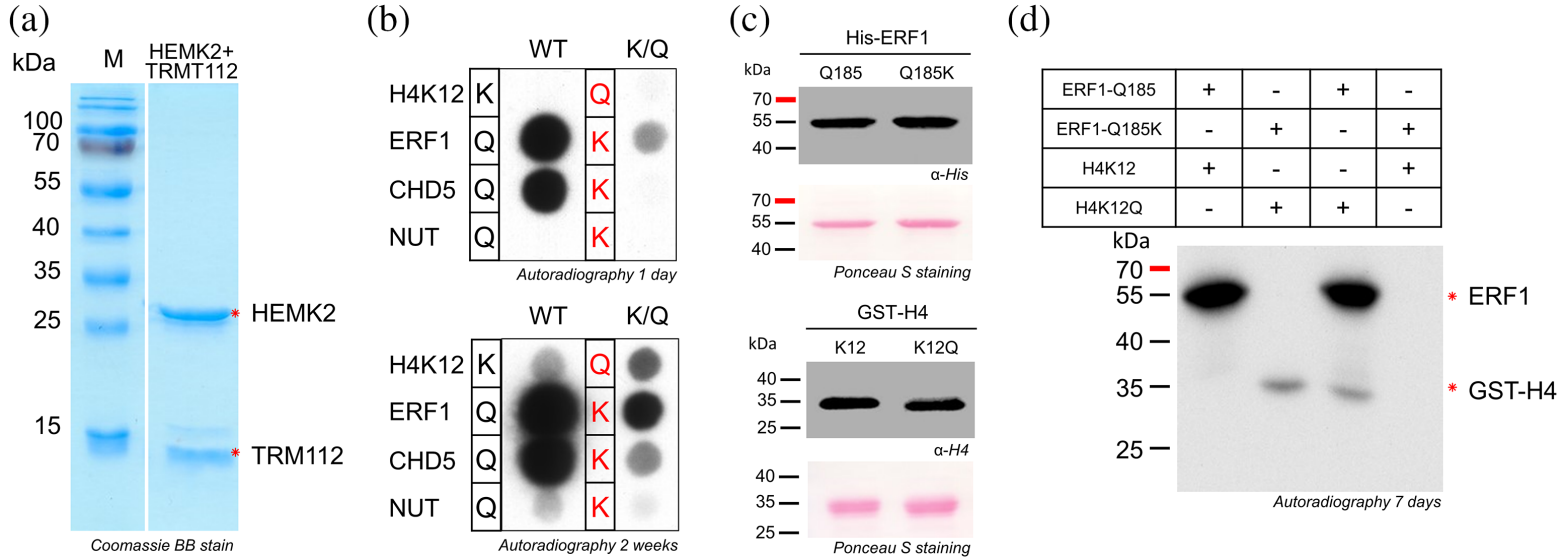
Purification and activity analysis of HEMK2 on different substrates at peptide and protein level. (a) Coomassie stained SDS gel of the purified HEMK2/TRMT112 complex. The HEMK2 and TRMT112 proteins are marked with red asterisk. (b) Methylation of peptide spot arrays containing the sequences of H4K12 (aa 6–19), ERF1 (aa 179–193) and as positive controls CHD5 (aa 1384–1398) and NUT (aa 1040–1054). In addition, the K‐to‐Q mutant of H4K12 and Q‐to‐K mutants of the other peptides were added (Table S1). Methylation was conducted using 1.09 μM HEMK2/TRMT112 for 1 h. (c) Ponceau S staining and western blot analysis of the His‐tagged ERF1‐Q185/Q185K or GST‐tagged H4K12/K12Q proteins using His or H4 antibodies, respectively, to show equal loading. (d) The ERF1‐Q185/Q185K (3.1 μM) and GST‐H4K12/K12Q proteins (1.5 μM) were incubated overnight in different combinations with HEMK2/TRMT112 (2.8 μM) in presence of radioactively labeled [methyl‐^3^H]‐AdoMet. In panels b and d, the methyl group transfer was detected by autoradiography after indicated time points.

Previous studies from our lab used substrate specificity analysis to determine the preferred sequence motif G‐Q‐X_3_‐R of HEMK2 for Q methylation in ERF1 (Kusevic et al., 2016). Comparison of this motif with the sequence of H4K12 shows a related G‐K G G A K motif with a G at the −1 and K at the +4 site. Hence, the question arose if the amino acid sequence recognition of HEMK2 for Q‐ and K‐methylation is similar. Therefore, the His‐tagged HEMK2 together with the untagged complex partner TRMT112 were recombinantly expressed in *E. coli* and isolated with good yield and purity (Figure 1a). SPOT peptide arrays were synthesized on a cellulose membrane containing 15 aa long peptides of the H4K12 (aa 6–19) and ERF1‐Q185 sequences (aa 179–193) with the respective K12 or Q185 in the center. In addition, the H4K12Q and ERF1‐Q185K sequences were included. As positive controls, the sequences of the validated HEMK2 non‐histone substrates CHD5 (aa 1384–1398) and NUT (aa 1040–1054) (Kusevic et al., 2016) and also their Q‐to‐K mutations were synthesized on the same array (Table S1). The peptide arrays were incubated with HEMK2/TRMT112 in presence of radioactively labeled [methyl‐^3^H]‐AdoMet and the methyl group transfer was detected by autoradiography. After 1 day of film exposure, a strong methylation signal was observed for ERF1‐Q185 and CHD5‐Q1390. A lower methylation signal appeared for the ERF1‐Q185K and CHD5‐Q1390K. After longer film exposure, methylation of H4K12 and NUT‐Q1046 became visible as well. Mutation of H4K12 to Q led to an increase in the methylation signal, but the H4K12Q methylation still remained weaker than that of ERF1‐Q185K. In summary, with all peptides, methylation of the glutamine residues resulted in a much stronger methylation signal than methylation of lysine residues. When comparing Q‐ and K‐methylation in different sequence contexts, ERF1 clearly was the most preferred target sequence and H4 was disfavored (Figure 1b).

### 2.2 HEMK2 prefers methylation of Q over K at protein level

Since methylation of protein substrates can differ from peptide substrates due to the target site accessibility and interaction with 3D epitopes in folded proteins, we next compared methylation of ERF1 and H4 by HEMK2 at protein level. To this end, full‐length histone H4 was cloned as GST‐fusion protein, while His‐tagged ERF1 was taken from our previous study (Kusevic et al., 2016). The Q‐to‐K or the K‐to‐Q mutants were introduced by site‐directed mutagenesis. For methylation, equal amounts of ERF1 and H4 WT or mutated proteins (Figure 1c) were mixed in different combinations and methylated by HEMK2/TRMT112 to allow a direct comparison of the preferences of HEMK2 for both proteins. Thereafter, the methylated samples were separated by SDS‐PAGE and the methyl group transfer was detected by autoradiography. In Figure 1d, the autoradiography image after 7 days of exposure is shown. A strong methylation of ERF1‐Q185 was observed, but no signal at the expected size of H4K12 was detected. With the H4K12Q mutant, a methylation signal was observed, while the ERF1‐Q185K mutation resulted in the loss of the methylation signal. Comparison of the methylation signals obtained when both proteins contain a glutamine residue at the target position, showed that the methylation of ERF1 was much stronger than that of H4K12. Methylation of both targets was lost if lysine was present at the target position. These results were reproduced by performing protein methylation experiments using ERF1‐Q185, ERF1‐Q185K, H4K12 and H4K12Q in individual methylation reactions (Figure S1). Overall, the protein methylation data were consistent with the peptide array results, showing a methylation preference of HEMK2 for Q at the target position, and a sequence preference for ERF1 over H4K12. Of note, experiments presented later in full detail showed that after using a 5‐fold higher amount of the H4 protein and longer film expose, a weak H4K12 methylation signal was detected (Figure 6a) indicating that HEMK2 methylates H4K12 under our conditions, but the methylation is very weak.

### 2.3 Specificity analysis of HEMK2 using different template sequences

As shown in Section 2.2, the ERF1 sequence was preferred over H4K12 although the latter also contained the G‐Q/K‐X_3_‐R related G‐Q/K‐X_3_‐K motif. These findings suggest that the target sequence interaction for Q‐ and K‐methylation could be different. To investigate this hypothesis, SPOT peptide arrays were synthesized on a cellulose membrane using the ERF1‐Q185 and H4K12 substrate sequence as template including peptides with all possible single amino acid exchanges (except cysteine or tryptophan) at all positions of the template sequence. Methylation of the SPOT arrays by HEMK2/TRMT112 revealed the relative preferences of the enzyme for each peptide, allowing to determine a preference profile for each amino acid residue at each position in the substrate sequence. To reproduce these results, methylation of a set of corresponding arrays in which the template sequence was randomized only from position −3 to +3 (Figure S2) was performed.

**FIGURE 2:**
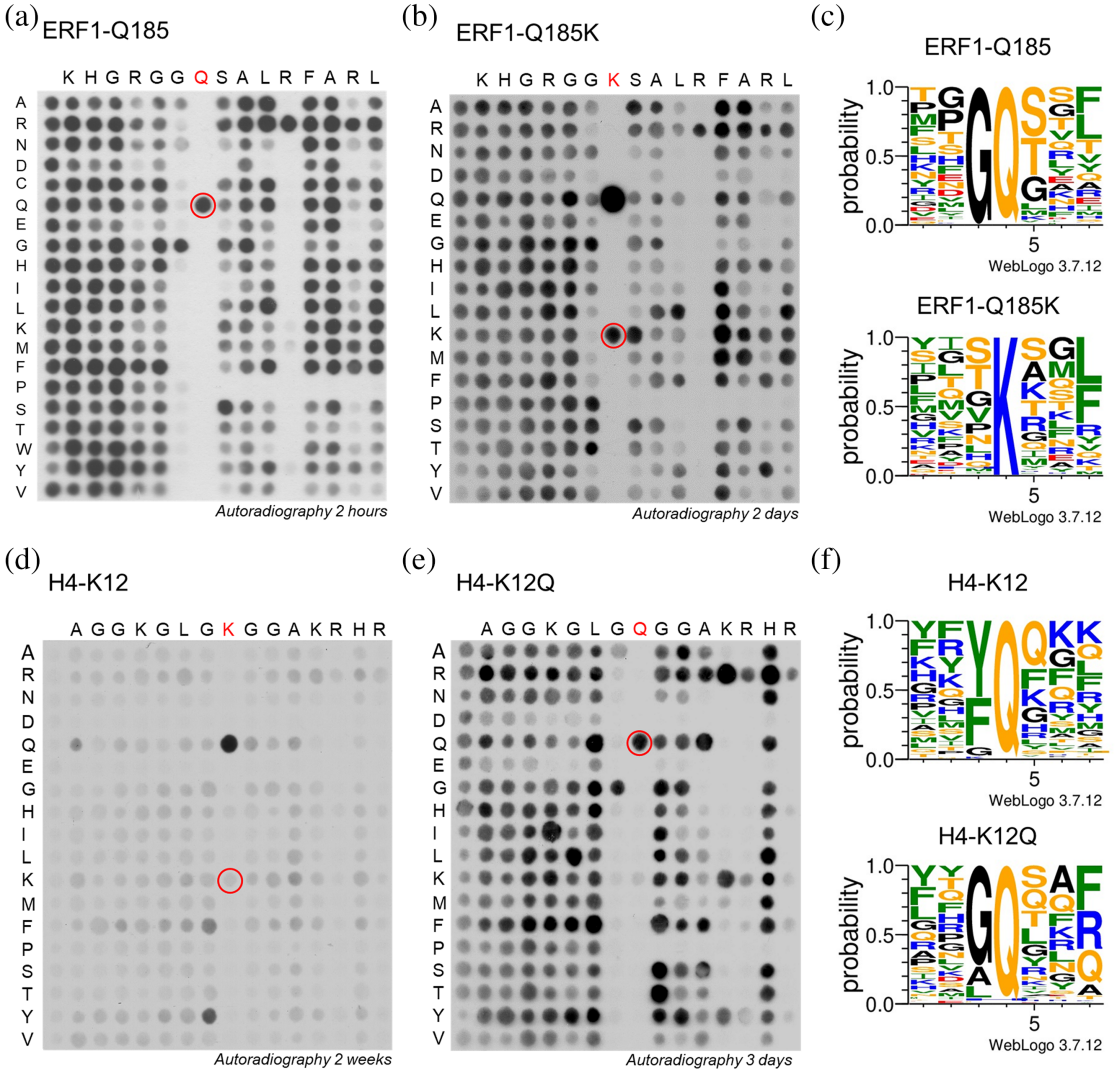
Substrate specificity analysis of HEMK2 using peptide SPOT arrays. Arrays of 15 aa long peptides were synthesized using different template sequences as represented in the horizontal axis. Each residue was systematically exchanged against other amino acid residues (except C and W in most cases) as shown in the vertical axis. For methylation, the membranes were incubated with HEMK2/TRMT112 in the presence of radioactively labeled AdoMet and the transfer of methyl groups was visualized by autoradiography after indicated film exposition times. (a) Template sequences of ERF1 (amino acid 179–193) with the target Q185 in the center. Image taken from (Kusevic et al., 2016) but reanalyzed. (b) Template sequences of ERF1‐Q185K (amino acid 179–193) with K185 in the center. (c) Weblogo of the preference profiles obtained with ERF1 based on the data in panels a and b and Figure S2. Prepared using WebLogo 3 (http://weblogo.threeplusone.com/). (d) Template sequences of H4K12 (amino acid 6–19) with the target K12 in the center. (e) Template sequence of H4K12Q (amino acid 6–19) with Q12 in the center. (f) Weblogo of the preference profiles obtained with H4K12 based on the data in panels d and e and Figure S2. Prepared using WebLogo 3 (http://weblogo.threeplusone.com/). Methylation reactions in panel b, d and e were conducted using 1.8 μM HEMK2/TRMT112 for 2 h.

Using the ERF1‐Q185 template sequence (Figure 2a, Figure S2a), the methylated arrays showed high preference for G at the −1 position and R at position +4 as observed previously (Kusevic et al., 2016). P residues are not tolerated at the positions on the C‐terminal side (+side) of the target Q. Strikingly, in the context of the ERF1‐Q185K template sequence (Figure 2b, Figure S2a), the reversion of the Q185K back to Q at the target position led to a very strong methylation signal. For better quantitative analysis, the spots representing the “backmutation” of Q at the target site in the ERF1‐Q185K and H4K12Q template sequences, were not included in the reproduction arrays (Figure S2) to avoid the generation of very strong signals at these spots. Analysis of the ERF1‐Q185K array methylation data shows that the preference for G at the −1 position was lost and S and T were preferred at this site (Figure 2a–c). Additionally, P was also accepted albeit more weakly. In contrast, other preferences in ERF1‐Q185K methylation did not differ from Q‐methylation, because still only R is accepted at position +4 and P is not tolerated on +side of the target residue.

In the corresponding peptide array methylation experiment with the H4K12 template sequence (Figure 2d, Figure S2b), overall very weak methylation was detected. The mutation of K12 to glutamine led to a massive increase in the methylation signal, again illustrating the strong preference of HEMK2 for glutamine methylation. In addition, an exchange of the natural G11 against Y led to a strong increase in methylation. A similar but weaker stimulation was also observed for the G11F exchange indicating that lysine methylation by HEMK2 is favored with a Y (or to lower extent F) present at the −1 position. These findings document that the H4 sequence is not an optimal for K12 methylation by HEMK2. Beyond that, the H4K12 peptide array methylation did not provide much information regarding sequence specific preferences, most likely due to the fact that methylation levels are weak and the H4 template sequence is not preferred for lysine methylation.

The specificity analysis of H4K12Q (Figure 2e,f, Figure S2b) revealed very similar results as the ERF1‐Q185 analysis, including the very clear preference for G at position −1. At position +4, the H4K12 template sequence contains a K, but the specificity profile shows that R is even more preferred at this place similar to the preference for R at the +4 site in ERF1‐Q185. The preference for R over K at the +4 site could be one reason for the lower activity of HEMK2 in the H4K12 sequence context. Interestingly, R/K is also preferred at the +5 site in the case of K‐methylation. AT the +side of the K12Q position, P is strongly disfavored. These results indicate that the amino acid sequence preferences of HEMK2 for Q methylation are similar regardless if the natural ERF1‐Q185 or artificial H4K12Q sequence is used as template.

### 2.4 Identification of additional HEMK2 substrates for K‐methylation

**FIGURE 3:**
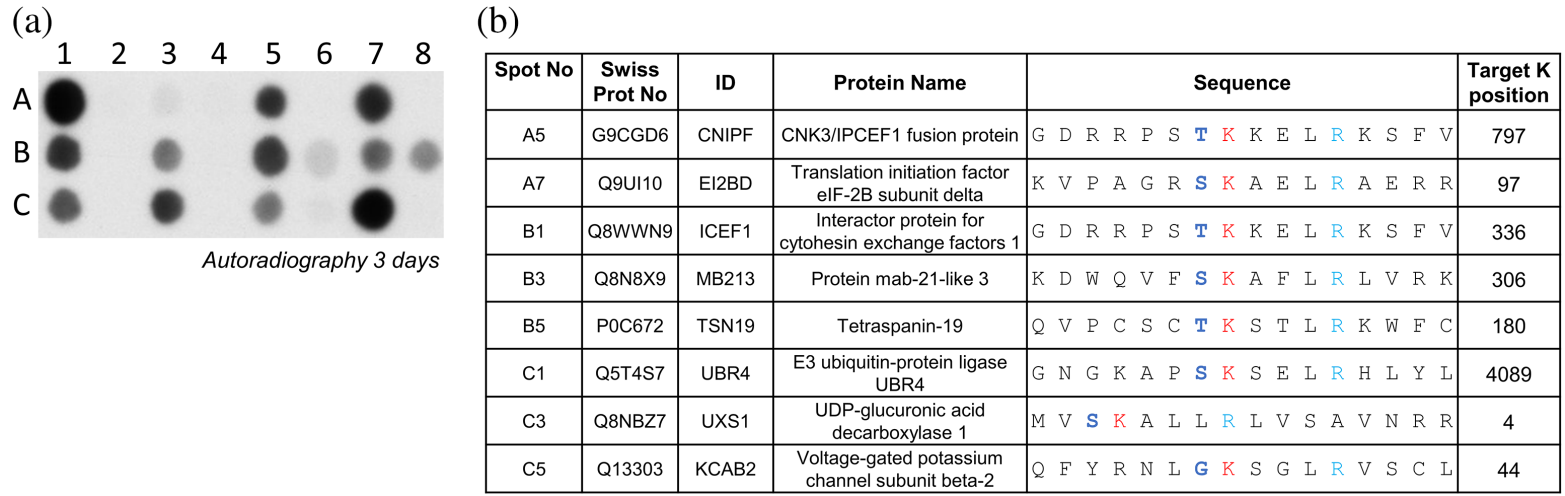
Identification of additional HEMK2 lysine substrates. (a) Based on the peptide array methylation experiment shown in Figure S3, the nine strongest new HEMK2 K‐methylation substrates were selected and further analyzed together with their K‐to‐A mutants. As controls, ERF1‐Q185K (Spot A1/C7), H4K12 (Spot A3) and ERF1‐Q185A (Spot A2/C8), and H4K12A (Spot A4) was included (Table S3). Methylation was conducted using 1.6 μM HEMK2/TRMT112 for 1 h. (b) Sequence and protein information of the eight specifically methylated peptides.

We have shown in Section 2.3 that HEMK2 K‐methylation preferentially occurs in a −1 site sequence context which is different from the context preferred for Q‐methylation, but so far strong K‐methylation was only detected with the non‐natural ERF1‐Q185K peptide. To find out if natural K‐methylation targets of HEMK2 can be identified, we used the relaxed K‐methylation profile determined here (allowing for G, S, T and P at the −1 site) and searched for potential substrate proteins in the human proteome using Scansite 4.0 (Obenauer et al., 2003). One hundred and fifty‐two putative substrate proteins were selected for methylation analysis and corresponding 15 aa long peptides with the target‐K in the center were synthesized on a SPOT array and methylated by HEMK2/TRMT112. As positive controls ERF1‐Q185K (Figure S3 Spot A1/H17) and H4K12 (Figure S3 Spot A3/H19), as well as their corresponding K‐to‐A mutants (Figure S3 Spot A2/H18 and A4/H20) were included (Table S2). From this array, the nine peptides with the strongest signals were selected and synthesized on an additional array with their corresponding K‐to‐A mutants (Table S3). As shown in Figure 3, among them eight showed specific methylation signals at the expected target lysine within the predicted S/T‐K‐X_3_‐R motif, four of them in comparable intensity as methylation of the ERF1‐Q185K peptide. Of note, all four strong targets and seven out of eight targets that were specifically methylated contain S or T at the −1 site, confirming the previous data that these residues are most preferred in K‐methylation. In contrast, the R/K preference at the +5 site observed in the ERK1 K‐methylation array apparently is less important in the context of other peptide sequences.

### 2.5 Structural interpretation of the HEMK2 sequence preferences

**FIGURE 4:**
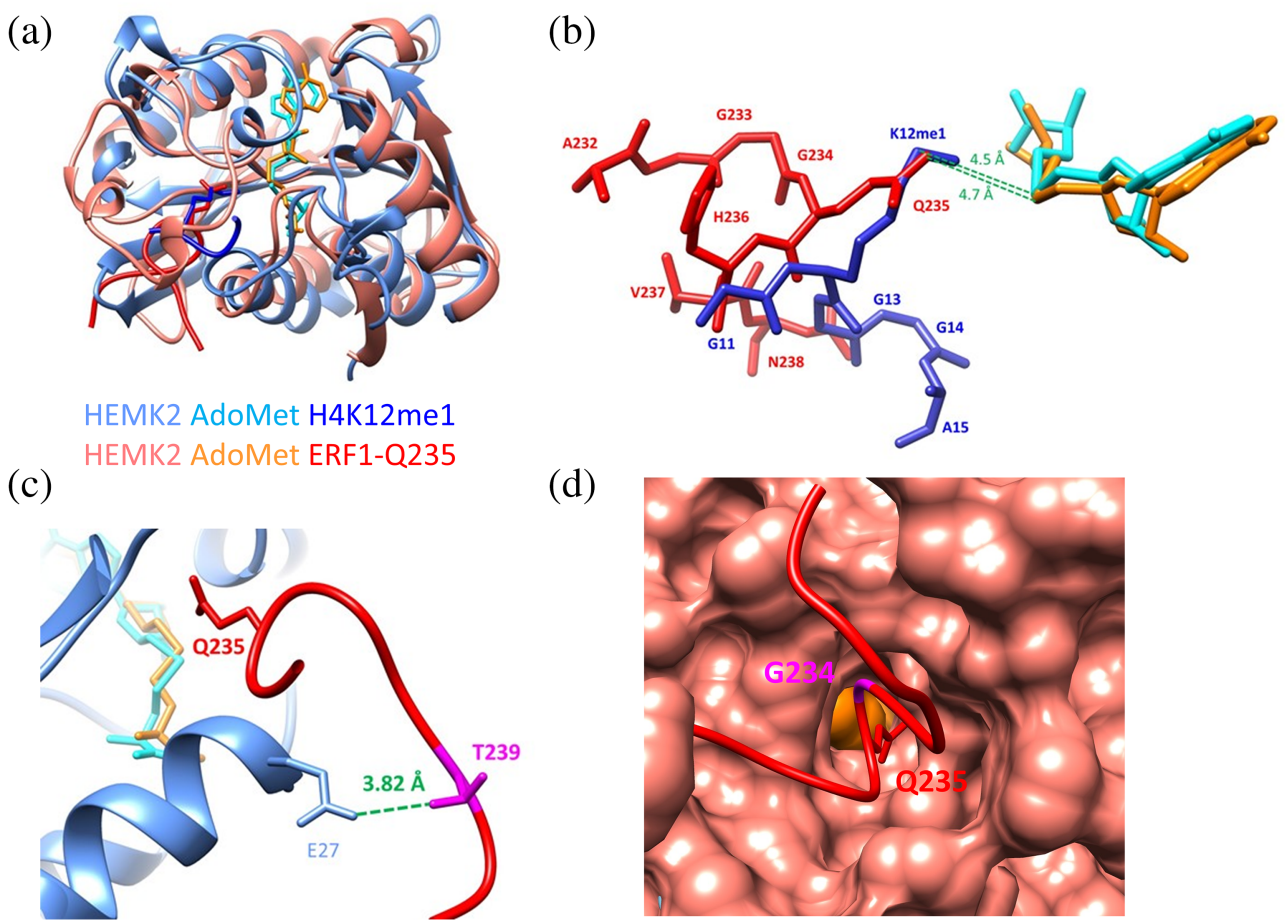
Superposition of the human HEMK2 in complex with H4K12me1 (pdb 6H1E) (Metzger et al., 2019) with the bacterial HEMK2 with bound bacterial RF1 (pdb 2B3T) (Graille et al., 2005). (a) Overview of the proteins. Human and bacterial HEMK2 are shown in pale blue and red, respectively. The H4K12 peptide and a part of RF1 containing the target‐Q235 are shown in blue and red, the corresponding AdoMet molecules in cyan and orange, respectively. (b) Detail of the superposition showing the target peptide/protein region and AdoMet molecules. (c) Detail of the superposition showing the RF1 target region and parts of the human HEMK2. RF1‐T239 (corresponding to an R in human ERF1) is shown in pink. (d) Detail of the superposition showing the RF1 target region with RF1‐G184 colored in pink. The human HEMK2 is shown in surface representation in red.

There are two structures of HEMK2 enzyme–substrate complexes available, one of them showing the *E. coli* HemK2 enzyme bound to the *E. coli* RF1 translation termination factor containing Q235 as target residue (pdb 2B3T) (Graille et al., 2005). The second structure shows the human enzyme bound to H4 containing K12 as target residue (pdb 6H1E) (Metzger et al., 2019). Both protein structures superimpose very well with an RMSD of 1.05 Å for the Cα‐atoms although the N‐terminal parts of the proteins are not conserved (residue 1–89 of the human enzyme) (Figure 4a). After manual adjustment of the Q235 Cγ‐Cδ dihedral angle, the target nitrogen atoms (Q235 Nε and K12 Nζ) are placed very close to each other in the superimposed structures with a distance of 0.6 Å and at distances of 4.7 Å (Q235) and 4.5 Å (K12) to the AdoHcy S‐atom (Figure 4b). HemK2 binds the RF1 peptide in a closed hairpin conformation forming extensive interactions with the protein (Figure 4b). The tight hairpin conformation could explain why P is not tolerated at the +side positions. At the +4 position, an R is strongly preferred by human HEMK2, but the *E. coli* RF1 substrate protein carries a T (T239) at this site. This residue approaches the non‐conserved N‐terminal part of the HEMK2 protein. However, in the superimposed structures, RF1‐T239 closely approaches E27 of human HEMK2 with a distance of 3.82 Å (Figure 4c), which could explain the strong preference for R resides at the +4 site in human HEMK2. At the −1 position, a G is strongly preferred by human HEMK2. This residue is bound in the highly conserved active site region in a very narrow pocket, explaining the high preference for G being the smallest amino acid at this site (Figure 4d).

The peptide conformation in the H4K12 complex differs markedly from that of the HemK2‐RF1 complex. First of all, despite the close approximation of the target N‐atoms described above, due to the longer side chain of K, the K12‐Cα atom is shifted by 2.1 Å away from the enzyme compared to the Q235‐Cα. Due to this, the G11 residue in the K12 peptide is not placed in the narrow −1 site pocket observed in the HemK2‐RF1 structure which could explain why a Tyr is highly preferred at this position for H4K12 methylation. Apart from the K12 itself, the H4 sequence does not provide many options for sequence specific contacts given the GLGKGGA sequence context. Consequently, in the H4K12 complex structure only the residues G11‐A15 are resolved. Our biochemical data show that an R at the +4 position is preferred for K12 methylation as well (similarly as in Q235 methylation) suggesting that in the productive conformation the +side regions of the Q235 and K12 peptides may adopt similar conformations. The results obtained with the ERF1‐Q185K and H4K12Q peptides will be interpreted in Section 3, as there are no direct structural data available for comparison.

### 2.6 Investigation of alternative PKMTs for H4K12 methylation

**FIGURE 5:**
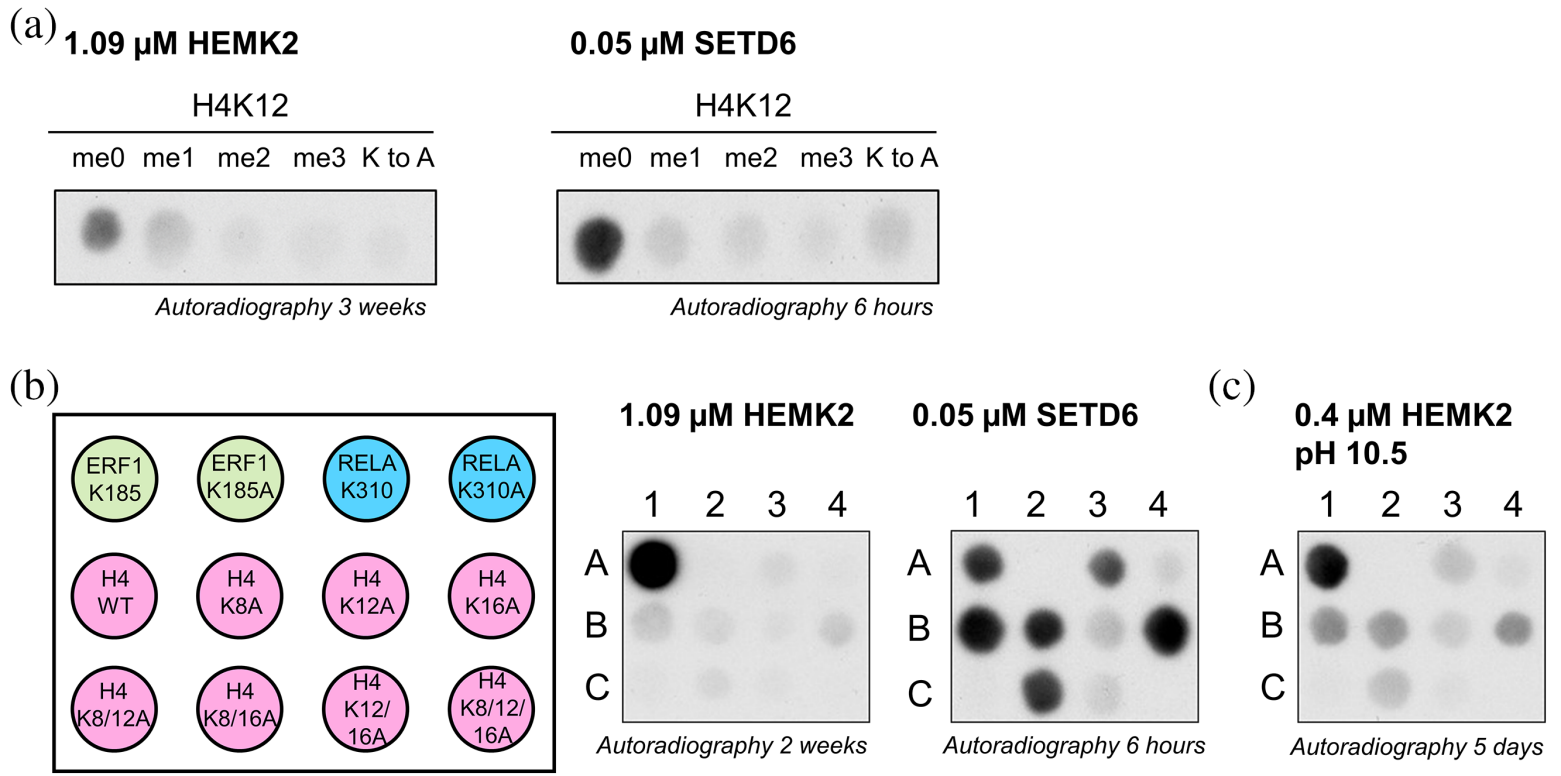
H4K12 peptide methylation activities of HEMK2 and SETD6. (a) Methylation of peptide arrays containing either unmethylated H4K12 or different methylated forms of H4K12, and H4K12A as negative control (Table S4), using 1.09 μM HEMK2/TRMT112 or 0.05 μM SETD6. Methyl group transfer was detected by autoradiography after indicated film exposition times. (b) Scheme of the peptides present on the peptide arrays shown in panels b and c. The arrays contain 15 aa long sequences of ERF1‐Q185K and RelA‐K310 with their corresponding K‐to‐A mutants in the first line. In the second line, the sequence of H4K12 and single mutations of K8, K12 and K16 to A were synthesized. The third line, contains all possible double mutations and the triple mutation of the K8/12/16 residues (Table S5). Peptide arrays were methylated with 1.09 μM HEMK2/TRMT112 or 0.05 μM SETD6 for 1 h. (c) The same peptide array was methylated with 0.4 μM HEMK2/TRMT112 at pH 10.5 for 2 h.

The data obtained so far indicate that HEMK2 prefers methylation of Q over K and the H4K12 does not provide an amino acid target sequence preferred by HEMK2. Consequently, the methylation of H4K12 peptide and protein by HEMK2 was very weak. Therefore, we tested several other PKMTs to investigate if an alternative enzyme could catalyze H4K12 monomethylation. A panel of different enzymes, NSD1 (Kudithipudi et al., 2014a), NSD2 (Khella et al., 2023), SMYD2 (Weirich et al., 2020), SET7/9 (Dhayalan et al., 2011), SETD6 (Kublanovsky et al., 2023), SET8 (Kudithipudi et al., 2012), SUV39H1 (Kudithipudi et al., 2017) and SUV39H2 (Schuhmacher et al., 2015) which are all well characterized in our lab, were overexpressed and purified by affinity chromatography. SDS‐PAGE analysis indicated good yield and quality for all of them (Figure S4a). To investigate the putative H4K12 methylation by these enzymes, small SPOT peptide arrays were synthesized with H4K12 peptides and the K12A variant as negative control (Figure S4b). The peptide arrays were methylated with the different enzymes mentioned above in presence of radioactively labeled [methyl‐^3^H]‐AdoMet and the methyl group transfer was detected by autoradiography. The peptide array methylation data revealed strong methylation of H4K12 and loss of methylation for the K12A peptide for SETD6. SETD6 is a well‐established lysine monomethyltransferase with a broad substrate spectrum (Levy et al., 2011; Feldman et al., 2019; Vershinin et al., 2016, 2021; Kublanovsky et al., 2023), with already documented activity on histone proteins H3 and H4 (Binda et al., 2013; Binda, 2020). To proof that SETD6 catalyzes monomethylation of the H4K12 peptide, arrays were synthesized with H4 peptides containing either unmethylated K12 or different methylated forms of K12 (Table S4). As shown in Figure 5a, SETD6 as well as HEMK2/TRMT112 only catalyze methylation of H4K12 using unmethylated H4K12 as substrate, but activity is lost with H4K12me1 indicating that they generate monomethylation. Next, we aimed to identify the target residue of SETD6 in the H4 (aa 6–19) peptide.

### 2.7 Analysis of H4K12 peptide methylation by HEMK2 and SETD6

Based on the result that SETD6 has a strong H4K12 methylation activity, we compared the methylation of H4K12 by HEMK2/TRMT112 or SETD6 in more detail using additional peptide arrays focusing on lysine methylation. The peptide array in Figure 5b contains in the first line peptide sequences of ERF1‐Q185K and RelA‐K310 with their corresponding K‐to‐A mutants. The ERF1 peptide serves as a positive control for HEMK2, while RelA is a known substrate for SETD6 (Levy et al., 2011). In the second line, the sequence of H4K12 and single mutations of K8, K12 and K16 to A were synthesized. The third line, represents all possible double K‐to‐A mutations and the triple mutation (Table S5). Peptide arrays were methylated with SETD6 and a 20 times higher concentration of HEMK2. As observed in the previous experiments, methylation with HEMK2/TRMT112 resulted in a strong methylation signal for ERF1‐Q185K and loss of methylation of ERF1‐Q185A confirming that methylation occurred at K185. Only a very weak methylation signal was observed for H4K12, but this signal was further reduced for the H4K12A peptide confirming a weak H4K12 methylation activity of HEMK2. In contrast, a strong methylation of ERF1‐Q185K, RelA‐K310 and H4K12 by SETD6 was detected accompanied by a loss of methylation of the methylation signal of the corresponding K‐to‐A peptides as expected. Methylation of the K‐to‐A mutant H4K12 peptides by SETD6 revealed strong loss of the methylation whenever K12 was mutated. The weak residual methylation of the K12A spots indicates that SETD6 has an additional, but much weaker, activity also at the K8 site.

Since it was observed that H4K12 methylation by HEMK2 is increased at higher pH, we repeated the peptide array methylation with slightly lower HEMK2 concentration and increased the pH to 10.5, the optimized pH for K‐methylation by HEMK2 determined previously (Woodcock et al., 2019). As shown in Figure 5c, the HEMK2 activity indeed increased, because the H4K12 methylation signal got stronger even after shorter film exposure of only 5 days. However, the overall preference of HEMK2 for ERF1‐Q185K methylation was still observed. Considering the signal intensities, enzyme concentrations, methylation and film exposition times, in can be estimated that SETD6 is >1000‐times more active than HEMK2 in H4K12 methylation at peptide level, even if the HEMK2 methylation is conducted at the optimized pH. For the continuation of our study, we considered that methylation reactions at pH 10.5 do not correspond to physiological conditions and this pH may lead to denaturation of target proteins. Therefore, we conducted the following in vitro protein methylation experiments at the previously established buffer conditions for HEMK2 and SETD6.

### 2.8 Analysis of H4K12 protein and nucleosome methylation by HEMK2 and SETD6

**FIGURE 6:**
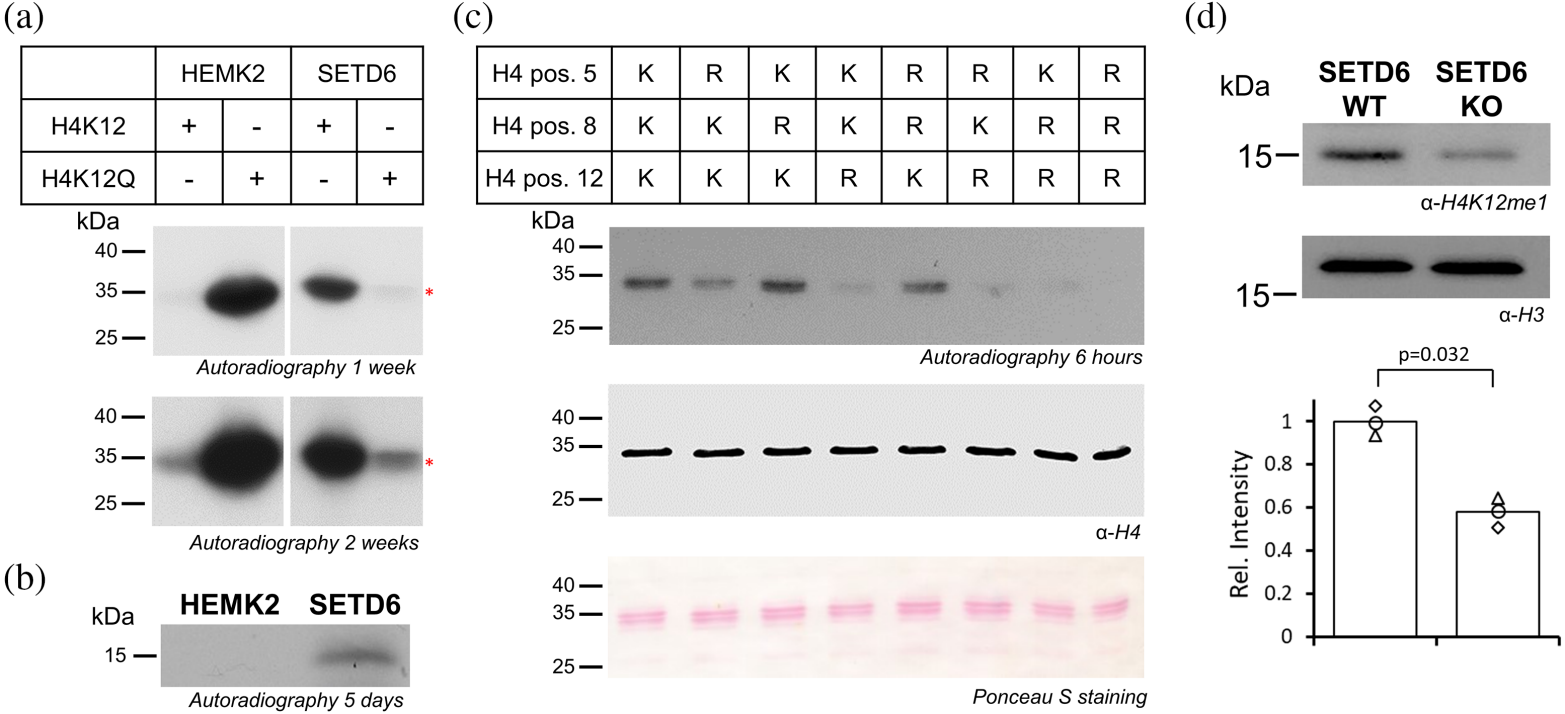
Comparison of H4K12 protein methylation activities of HEMK2 and SETD6. (a) SETD6 (2.5 μM) and HEMK2/TRMT112 (2.8 μM) were mixed with GST‐tagged H4 or GST‐tagged H4K12Q protein (7.5 μM) and incubated overnight in the presence of radioactively labeled [methyl‐^3^H]‐AdoMet. Methyl group transfer was detected by autoradiography after indicated film exposition times. (b) SETD6 (2.5 μM) and HEMK2/TRMT112 (2.8 μM) were mixed with recombinant nucleosomes and incubated overnight in the presence of radioactively labeled [methyl‐^3^H]‐AdoMet. Methyl group transfer was detected by autoradiography after indicated film exposition time. (c) Equal protein amounts (7.5 μM) of H4‐K5R, ‐K8R, ‐K12R, ‐K5R/K8R, ‐K5R/K12R, ‐K8R/K12R and ‐K5R/K8R/K12R, validated by Ponceau staining and western blot using the H4 antibody, were incubated with SETD6 (2.5 μM) for 3 h in the presence of radioactively labeled [methyl‐^3^H]‐AdoMet. The autoradiographic image shows methyl group transfer after 6 h. (d) Equal loading of histones isolated from DU145 SETD6 WT or KO was verified by Western blot analysis against histones H3. The presence of H4K12me1 was detected using the H4K12me1 specific antibody. The dots in the bar diagram show the H4K12me1 band intensities observed in three independent biological repeats. The bars represent the averages. The *p*‐value was determined by two flanked *t*‐test for paired data.

Next, the comparison of H4K12 methylation by SETD6 and HEMK2 was performed at protein level. In this case, roughly equal amounts of SETD6 and HEMK2/TRMT112 (Figure S5a) were mixed with GST‐tagged H4 or GST‐tagged H4K12Q protein and incubated in the presence of radioactively labeled [methyl‐^3^H]‐AdoMet. The autoradiographic image in Figure 6a shows strong methylation of GST‐H4 by SETD6, but very weak methylation by HEMK2. The K12Q mutation in the GST‐H4 construct led to strong methylation by HEMK2, but weak methylation with SETD6. In addition, recombinant nucleosomes were prepared and used in a similar methyltransferase reaction (Figure S5). The histone proteins were separated by Tricine gel electrophoresis and methylation detected by autoradiography. As shown in Figure 6b, a strong methylation signal of Histone H4 was observed after incubation with SETD6 but no methylation signal with HEMK2. These results further support the hypothesis that SETD6 could be the PKMT responsible for H4K12 monomethylation.

In Figure 6a, a weak SETD6 methylation signal was observed in the H4K12Q sample. This signal may result from the methylation of other lysine residues in this protein as already observed in the peptide arrays, because glutamine methylation by SETD6 has not been reported. To investigate if SETD6 can methylate other target lysine residues than K12 in H4, site‐directed mutagenesis was used to introduce K5R, K8R, K12R or all possible double mutations, and the corresponding triple mutation (Figure 6c). After cloning, the different constructs were overexpressed and purified. Equal protein amounts were used for methylation reactions performed as described above. After 6 h of film exposure, a clear methylation signal of H4 catalyzed by SETD6 was observed (Figure 6c). Strong reduction in methylation was visible in the H4‐K12R mutant and the double mutants containing the K12R mutation, but some weak methylation was still observed on these proteins, which was only lost completely in the K5R/K8R/K12R triple mutant. These data confirm that SETD6 mainly methylates H4K12, in addition to some much weaker activity at K5 and K8.

### 2.9 Analysis of H4K12 protein methylation in WT and SETD6 KO cells

So far, our data show that SETD6 catalyzes the methylation of H4K12 with high activity with peptide, protein and nucleosomal substrates in vitro. In the next step, we wanted to investigate if SETD6 is also involved in H4K12 methylation in human cells. For this, we used DU145 cells and a SETD6 KO DU145 cell lines, that have been described and validated previously (Kublanovsky et al., 2023). Histones were isolated from both cell lines and separated by SDS‐PAGE (Figure S6a). DU145 is a prostate cancer cell line, which was chosen as the original observation reporting H4K12 methylation by HEMK2/TRMT112 was also made in prostate cancer cells (Metzger et al., 2019). Equal loading of the histone extracts was verified by Western blot analysis against histones H3, and the H4K12 methylation status of histone H4 was detected using a H4K12me1 specific antibody (Figure 6d). Previously, the specific H4K12me1 binding of the antibody was validated by SPOT peptide array binding experiments and Western Blot analysis of the GST‐tagged H4 or H4K12R proteins methylated by SETD6 in presence of unlabeled AdoMet (Figure S6b,c). Strikingly, the Western blot analysis of H4 isolated from the SETD6 WT and KO DU145 cells showed that the H4K12me1 signal was strongly reduced for histone H4 isolated from the KO cells (Figure 6d). The experiment was conducted in three biological repeats. Intensity analysis of the bands in the individual Western blots demonstrated that the results are reproducible and highly significant (*p*‐value = 0.032, based on two‐tailed t‐test for paired samples). The residual H4K12me1 signal observed with H4 isolated from SETD6 KO cells could either indicate that other enzymes, like for example HEMK2, also contribute to H4K12 methylation to a lesser degree, or it could be due to antibody cross‐reactivity with other Kme1 sites on H4. Overall, our data obtained with the SETD6 KO DU145 human prostate cancer cells clearly indicate that SETD6 has a key role in the establishment of H4K12me1 in human cell line.

## 3 DISCUSSION

The HEMK2 protein methyltransferase has been described as PQMT and PKMT, which is a unique observation for this class of enzymes. We started this work by studying if the change in the nature of the target residue has consequences for the sequence specific interaction of this enzyme with its substrate peptide. We showed that Q‐methylation of the ERF1‐Q185 and H4K12Q peptides prefers G at the −1 site and R at the +4 site, both findings are in good agreement with structural (Graille et al., 2005; Metzger et al., 2019) and previous biochemical data (Kusevic et al., 2016). K‐methylation in the context of the ERF1‐Q185K peptide revealed a change in the sequence recognition with a preference for S and T at the −1 site, which can be explained by the movement of the peptide backbone away from the enzyme after incorporating the larger K‐side chain in the active site. Using the newly discovered sequence motif for K‐methylation of HEMK2, eight novel K‐methylation substrates derived from human proteins were identified. K‐methylation in the context of H4K12 was weak and our data showed that the H4K12 sequence does not provide a preferred sequence context for K‐methylation by HEMK2. Most strikingly, a Y is strongly preferred at the −1 site of K‐methylation in this sequence context.

While the peptide sequence preferences of ERF1‐Q185 and H4K12 methylation are well‐explained by the structural data, our data on ERF1‐Q185K and H4K12Q methylation can also be understood although no structural information is available for direct comparison. In case of ERF1‐Q185K complex, the larger K side chain is expected to cause a similar shift of the peptide backbone as observed in the K12 structure. However, the better contact potential of the ERF1 peptide to the HEMK2 active site, when compared with H4K12, may restrict this movement. This could explain that S/T are preferred over G at the −1 site for methylation of ERF1‐Q185K, which are larger than G, but Y (which is preferred in H4K12 methylation) is disfavored. Based on the ERF1‐Q185K methylation preferences, the +side part of the Q185K peptide is expected to adopt a similar conformation as the ERF1‐Q185 peptide. In case of the H4K12Q methylation, the higher methylation of this peptide is presumably explained by the peptide adopting a conformation closely resembling the ERF1‐Q185 peptide. This model is supported by the finding that the G at the −1 site is highly important for H4K12Q methylation which is a characteristic feature of Q‐methylation by HEMK2.

Next, we addressed the question of the target residue preferences. By using ERF1 and H4 peptides carrying Q or K at the target sites, we found that H4K12 methylation was very weak and determined the following order of catalytic activities: ERF1‐Q185 > ERF1‐Q185K > H4K12Q > > H4K12. These data indicate that Q is preferred over K as target residue and the ERF1 peptide sequence context is preferred over H4. However, a relatively strong K‐methylation was observed at the ERF1‐Q185K peptide in a non‐natural sequence context and in 8 newly discovered natural HEMK2 K‐methylation substrates. Additional experiments will be needed to investigate if these K‐methylation substrates are methylated at the protein level in cells and what is the regulatory role of Kme1 in these proteins.

Having observed the very low H4K12 methylation activity of HEMK2, we tested several other PKMTs for their ability to catalyze H4K12me1 and observed that the SETD6 PKMT is highly active in this reaction. Direct comparison of the H4K12me1 methylation activity of HEMK2 and SETD6 showed that SETD6 is >1000‐fold more active than HEMK2 at peptide and protein level. Methylation analysis of H4 proteins isolated from SETD6 WT and KO human DU145 cell lines showed strong reduction of H4K12me1 in H4 isolated from SETD6 KO cells confirming that SETD6 has an important role in H4K12 methylation in cells. Future studies will be needed to investigate the potential role of SETD6 mediated H4K12me1 methylation in chromatin regulation.

## 4 MATERIALS AND METHODS

### 4.1 Cloning of enzymes and substrate proteins

The bacterial expression vector pRSF‐Duet1 encoding the His‐tagged murine HEMK2 together with the untagged complex partner TRMT112 and the pRSET ERF1 vector was kindly provided by Dr. G. L. Xu (Liu et al., 2010). The full‐length protein of the human H4 was cloned C‐terminal to the GST‐tag in a pGex‐6p‐2 vector by Gibson assembly. The K‐to‐Q or Q‐to‐K mutation of H4K12 and ERF1, respectively, as well as H4‐K5R, ‐K8R, ‐K12R, ‐K5/8R, ‐K5/12R, ‐K8/12R, ‐K5/8/12R were introduced using a megaprimer PCR mutagenesis method (Jeltsch & Lanio, 2002). All cloning steps were confirmed by sequencing. The details and sources of the other PKMTs are as follows: The mouse NSD1 (aka KMT3B, amino acids 1700–1987 of UniProt 088491.1) and the GST‐tagged expression constructs of the human NSD2 (aka MMSET, amino acids 992–1240 of UniProt No O96028) were taken from (Kudithipudi et al., 2014a; Khella et al., 2023). The His‐tagged full‐length SET7/9 (aka KMT7, UniProt No Q8WTS6) expression construct was taken from (Dhayalan et al., 2011). The His‐tagged human SMYD2 (aka KMT3C, residues 1–433 of UniProt No Q9NRG4) was taken from (Weirich et al., 2020). The His_6_ SUMO‐tagged construct of human SETD6 (amino acids 17–449 of UniProt No. Q8TBK2‐2) was taken from (Kublanovsky et al., 2023). The GST‐tagged SET8 protein (aka KMT5A, amino acids 114–352 of UniProt No. Q9NQR1) was taken from (Kudithipudi et al., 2012). The GST tagged SUV39H2 (aka KMT1B, amino acids 112–410 of UniProt No. Q9H5I1) was taken from (Schuhmacher et al., 2015). The GST fused murine SUV39H1 SET domain (aka KMT1A, amino acids 82–412 of UniProt No. O54864) was taken from Kudithipudi et al. (2017).

### 4.2 Overexpression and purification of enzymes and proteins

For protein overexpression, the plasmids were transformed into *E. coli* BL21‐CodonPlus (DE3) cells (Novagen, USA) and grown in LB medium at 37°C until an OD_600_ of 0.6–0.8 was reached. The culture was then shifted to 20°C overnight (14–16 h) or 30°C for 4 h and protein expression was induced with 1 mM isopropyl‐D‐thiogalactopyranoside. Afterwards, the cells were harvested by centrifugation at 4500 rpm, washed once with STE buffer (10 mM Tris/HCl pH 8.0, 1 mM EDTA and 100 mM NaCl) and the cell pellet was stored at −20°C.

For purification of the GST‐tagged proteins, the cell pellet was thawed on ice, resuspended in sonication buffer (50 mM Tris/HCl pH 7.0, 150 mM NaCl, 1 mM DTT, 5% (w/v) glycerol) supplemented with protease inhibitor cocktail containing AEBSF‐HCL (1 mM, Biosynth), pepstatin (10 μM, Roth), aprotinin (0.4 μM, Applichem), E‐64 (15.14 μM, Applichem), leupeptin (22.3 μM Alfa Aesar) and bestatin (50 μM, Alfa Aesar) and lysed by sonication (14 rounds, 30% power, 4°C). Thereafter, the sample was centrifuged at 18,000 rpm for 90 min at 4°C and the supernatant was loaded onto a Glutathione Sepharose 4B resin (GE Healthcare) column, which was pre‐equilibrated with sonication buffer. Afterwards, the beads were washed once with sonication buffer and twice with washing buffer (50 mM Tris/HCl pH 8.0, 500 mM NaCl, 1 mM DTT, 5% (w/v) glycerol). Subsequently, the bound proteins were eluted with elution buffer (40 mM reduced glutathione, 50 mM Tris/HCl pH 8.0, 500 mM NaCl, 1 mM DTT, 5% (w/v) glycerol) and then dialyzed against low glycerol dialysis buffer I (20 mM Tris/HCl pH 7.4, 100 mM KCl, 0.5 mM DTT, 10% (w/v) glycerol) for 3 h and afterwards overnight against high glycerol dialysis buffer II (20 mM Tris/HCl pH 7.4, 100 mM KCl, 0.5 mM DTT, 60% (w/v) glycerol).

His‐tagged proteins were resuspended in 30 mL of sonification buffer (30 mM KPI, 500 mM KCl, 0.2 mM DTT, 1 mM EDTA, 20 mM imidazole, 10% glycerol), supplemented with protease inhibitor cocktail and lysed by sonication (14 cycles, 40 s per cycle). After the sonification, the sample was centrifuged at 18,000 rpm for 90 min at 4°C. The supernatant was transferred onto a Ni‐NTA agarose beads (Qiagen) purification column, which was pre‐equilibrated with sonification buffer. Afterward, the column was washed three times with sonification buffer. For elution of the bound proteins, elution buffer (30 mM KPI, 500 mM KCl, 0.2 mM DTT, 1 mM EDTA, 220 mM imidazole, 10% glycerol) was added. The eluted proteins were dialysed for 3 h in dialysis buffer I (20 mM HEPES, 200 mM KCl, 0.2 mM DTT, 1 mM EDTA, 10% glycerol) followed by dialysis buffer II (20 mM HEPES, 200 mM KCl, 0.2 mM DTT, 1 mM EDTA, 65% glycerol) overnight. The purified sample was then stored at −20°C. The purified proteins were analyzed by sodium‐dodecyl‐sulfate‐polyacrylamide gel electrophoresis (SDS‐PAGE).

### 4.3 Peptide array synthesis

Synthesis of the peptide arrays was performed with the SPOT synthesis method (Frank, 2002) using an Autospot Multipep synthesizer (Intavis AG). Each spot contained approximately 9 nmol peptide (Autospot Reference Handbook, Intavis AG) and the successful synthesis of the peptides on the cellulose membrane was qualitatively confirmed by bromophenol blue staining (Kudithipudi et al., 2014b; Weirich & Jeltsch, 2022). Peptide arrays were prepared using the sequences of H4K12 (amino acid 6–19, with an additional A at the N‐terminus to place K12 in the center and avoid starting the peptide with a K) and ERF1 (amino acid 179–193) as template sequence with the respective Lys (Yang et al., 2004) or Gln^185^ in the center. Arrays based on H4K12Q and ERF1‐Q185K were prepared accordingly. The peptide sequences of all SPOT arrays are specified in Tables S1–S5. Scansite 4.0 searches (https://scansite4.mit.edu/) were restricted to human proteins otherwise using default settings (Obenauer et al., 2003). Weblogos were prepared using WebLogo 3 (http://weblogo.threeplusone.com/).

### 4.4 Peptide array methylation

The peptide arrays were pre‐incubated in protein specific methylation buffer (HEMK2/TRMT112: 10 mM Tris/HCl pH 7.6, 50 mM KCl, 10 mM Mg/OAc, 1 mM DTT; NSD1/NSD2/SMYD2/SUV39H1/SUV39H2: 50 mM Tris/HCl pH 9, 5 mM MgCl_2_, 4 mM DTT; SET7/9: 20 mM Tris/HCl pH 9, 5 mM DTT; SETD6: 50 mM Tris/HCl pH 9, 5 mM DTT; SET8: 20 mM HEPES pH 8, 50 mM NaCl, 5 mM DTT) on a shaker for 5 min at room temperature. After this pre‐incubation step, the peptide arrays were methylated in methylation buffer containing the different PKMTs at concentrations mentioned in the text in the presence of 0.76 μM labeled [methyl‐^3^H]‐AdoMet (Perkin Elmer Inc., dissolved at 25 μM in 10 mM sulfuric acid) at room temperature. Afterwards, the peptide arrays were washed 5 times for 5 min each in 100 mM NH_4_HCO_3_ and 1% SDS and 5 min in Amplify NAMP 100 V from GE Healthcare. Once complete, arrays were exposed to Hyperfilm™ high‐performance autoradiography films (GE Healthcare) in the dark at −80°C. Film development was performed with an Optimus TR developing machine.

### 4.5 Histone purification and nucleosome reconstitution

Histone purification, octamer formation and nucleosome reconstitution was performed as previously described (Khella et al., 2023; Bröhm, 2022) Histone octamers were analyzed and purified by size exclusion chromatography (Figure S5b). The DNA assembly with the histone octamer in the reconstituted nucleosomes was validated by electromobility gel shift assays (Figure S5c). The histone protein content of the reconstituted nucleosomes was validated by SDS‐gel electrophoresis (Figure S5d).

### 4.6 Protein and nucleosome methylation assay

His‐tagged ERF1‐WT/Q185K and GST‐tagged H4 WT/K12Q proteins or recombinant nucleosomes were incubated with HEMK2/TRMT112 or SETD6 in methylation buffer supplemented with 0.76 μM labeled [methyl‐3H]‐AdoMet (Perkin Elmer) at 25°C. Concentrations and methylation times are stated in the text. The methylation reactions were stopped by the addition of SDS loading buffer and boiling for 5 min at 95°C. Equal loading of target protein amounts was confirmed by Coomassie Brilliant Blue staining and Western Blot using as primary antibody anti‐H4 (Millipore, 05‐858, Lot: 3768156) for GST‐tagged H4 or anti‐His (Qiagen, Hilden, Germany) for His‐tagged ERF1. The methylated protein samples were separated by 16% SDS‐PAGE, whereas methylated nucleosome samples were separated by tricine gel electrophoresis. Then, the gels were incubated for 1 h in Amplify NAMP 100 V (GE Healthcare) and dried for 90 min at 70°C under vacuum. The dried gels were exposed to Hyperfilm™ high‐performance autoradiography films (GE Healthcare) in the dark at −80°C. Film development was performed with an Optimus TR developing machine.

### 4.7 Antibody validation

The H4K12me1 antibody (PTM BIO, PTM‐685RM) used in the western blot analysis was validated by peptide array binding. The peptide array consisted of four spots corresponding to four different K12 methylation states of the H4K12 peptide (unmodified, H4K12me1, H4K12me2, and H4K12me3) and as negative control one additional spot with H4K12A was included. After blocking with 5% milk in 1xTBST buffer, the array was incubated with the primary antibody solution for 1 h at room temperature followed by washing three times. Then, the array was kept in secondary antibody solution (anti‐rabbit HRP; NA934V, GE Healthcare) for 1 h. After washing again, the signal was detected by chemiluminescence after the addition of Pierce ECL Western Blotting substrate. In addition, the GST‐tagged H4 protein or the GST‐tagged H4K12R protein was incubated with SETD6 in the presence of unlabeled AdoMet under same methylation conditions as described above. The methylation signal was detected by western blot with the H4K12me1‐specific antibody (PTM BIO, PTM‐685RM) as described.

### 4.8 Cell cultivation and histone extraction

SETD6 WT and KO DU145 cells were prepared as described (Kublanovsky et al., 2023). Histones were isolated by the acid extraction method as described by Shechter et al. (2007). Equal loading was validated by western blot analysis using the primary antibodies against H3 (ab10799, Abcam) and H4K12 methylation was detected using the H4K12me1 antibody (PTM BIO, PTM‐685RM) as described in Section 4.7. The signal was visualized by chemiluminescence after the addition of Pierce ECL western Blotting substrate.

## AUTHOR CONTRIBUTIONS

**Sara Weirich:** Conceptualization; investigation; validation; methodology; formal analysis; writing – original draft; writing – review and editing; visualization. **Gizem T. Ulu:** Investigation; validation; formal analysis; writing – review and editing; methodology; visualization. **Thyagarajan T. Chandrasekaran:** Investigation; validation; formal analysis; writing – review and editing. **Jana Kehl:** Investigation; validation. **Jasmin Schmid:** Investigation; validation. **Franziska Dorscht:** Investigation; validation; formal analysis; writing – review and editing. **Margarita Kublanovsky:** Investigation; validation; methodology; formal analysis; resources. **Dan Levy:** Supervision; resources; funding acquisition; writing – review and editing; project administration. **Albert Jeltsch:** Funding acquisition; conceptualization; supervision; formal analysis; visualization; writing – review and editing; writing – original draft; project administration.

## FUNDING INFORMATION

This work was supported by a joint grant of the DFG to AJ and DL (JE 252/38‐1). DL was also supported by The Israel Science Foundation (285/14 and 262/18), The Israeli Cancer Research Foundation Israel (ICRF), and from the Israel Cancer Association.

## CONFLICT OF INTEREST STATEMENT

The authors declare that they have no conflict of interest.

## Supporting information


**Appendix S1:** Supporting Information
