# Supplementary material for: Distinct specificities of the HEMK2 protein methyltransferase in methylation of glutamine and lysine residues
Source: Protein Sci. 2024 Jan 23;33(2):e4897. doi: 10.1002/pro.4897 (PMC10804810; doi:10.1002/pro.4897)
Supplement: Supplementary file 1 — Appendix S1: Supporting Information [file PRO-33-e4897-s001.pdf]

# Distinct specificities of the human HEMK2 protein methyltransferase in methylation of glutamine and lysine residues

Sara Weirich#, Gizem T. Ulu#, Thyagarajan T. Chandrasekaran, Jana Kehl, Jasmin Schmid, Franziska Dorscht, Margarita Kublanovsky, Dan Levy & Albert Jeltsch*

## Supplemental information

### Supplemental Figures

Supplemental Figure 1: Activity analysis of HEMK2 on different substrates at protein level.

Supplemental Figure 2. Substrate specificity analysis of HEMK2/TRMT112.
Supplemental Figure 3. Search for additional HEMK2 peptide substrate candidates.
Supplemental Figure 4. Purification and activity analysis of different PKMTs on the H4K12 peptide.

Supplemental Figure 5. Loading controls and experiments for recombinant H3.1 nucleosome formation and methylation.
Supplemental Figure 6. Loading controls and H4K12me1 antibody validation.

### Supplemental Tables

Supplemental Table 1: Sequences of the peptide spots shown in Figure 1B.

Supplemental Table 2: Sequences of the peptide spots shown in Supplemental Figure 3.

Supplemental Table 3: Sequences of the peptide spots shown in Figure 3A.

Supplemental Table 4: Sequences of the peptide spots shown in Figure 5A.

Supplemental Table 5: Sequences of the peptide spots shown in Figure 5B and C.

## Supplemental Figures

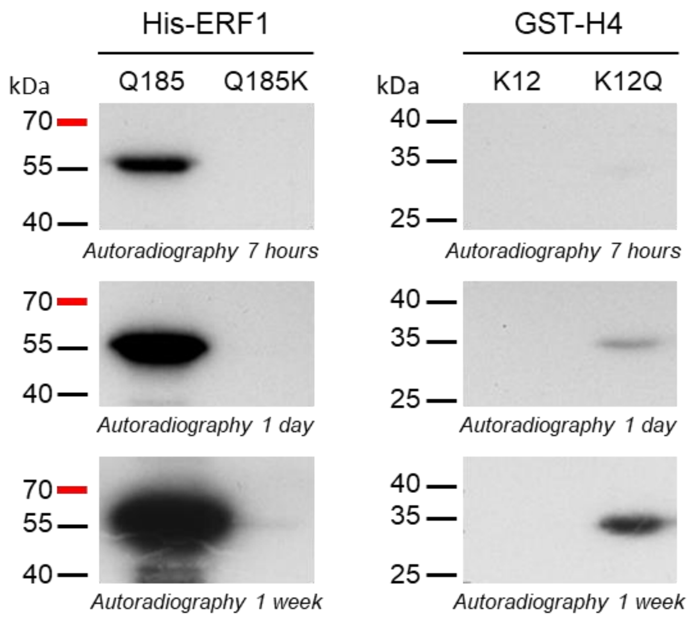


**Supplemental Figure 1: Activity analysis of HEMK2 on different substrates at protein level.** The ERF1-Q185/Q185K (3.1 µM) or H4K12/K12Q (1.5 µM) proteins were incubated overnight with HEMK2/TRMT112 (2.8 µM) in presence of radioactively labelled [methyl-$^{3}$H]-AdoMet. Methyl group transfer was detected by autoradiography after indicated film exposition times.

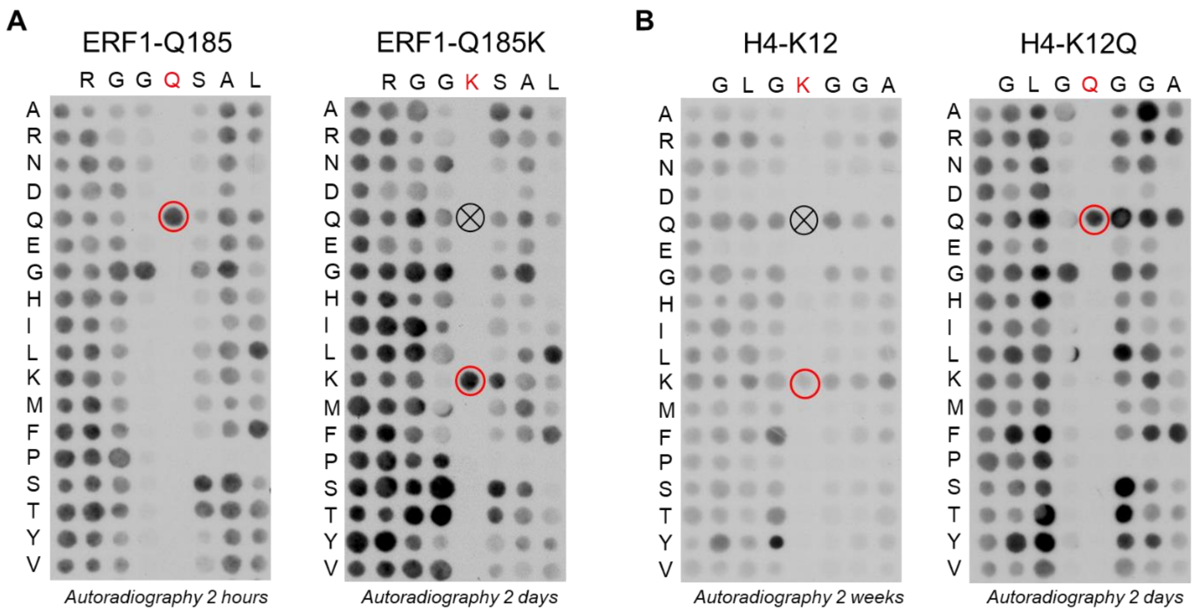


**Supplemental Figure 2. Substrate specificity analysis of HEMK2/TRMT112.** Smaller substrate specificity arrays containing 15 amino acid long peptides with the template sequence randomized only from position -3 to +3. In this region, the template sequence residues were exchanged by all proteinogenic amino acids (except C and W). **A)** Template sequences of ERF1 (aa 179-193) with the Q/K$^{185}$ in the center. **B)** Template sequences of H4K12 (aa 6-19) with the K/Q$^{12}$ in the center. The spots representing the sequences of ERF1-Q185 and H4K12Q were not included to avoid oversaturation by Q methylation, indicated by crossed circles. Methyl group transfer was detected by autoradiography after indicated film exposition times.

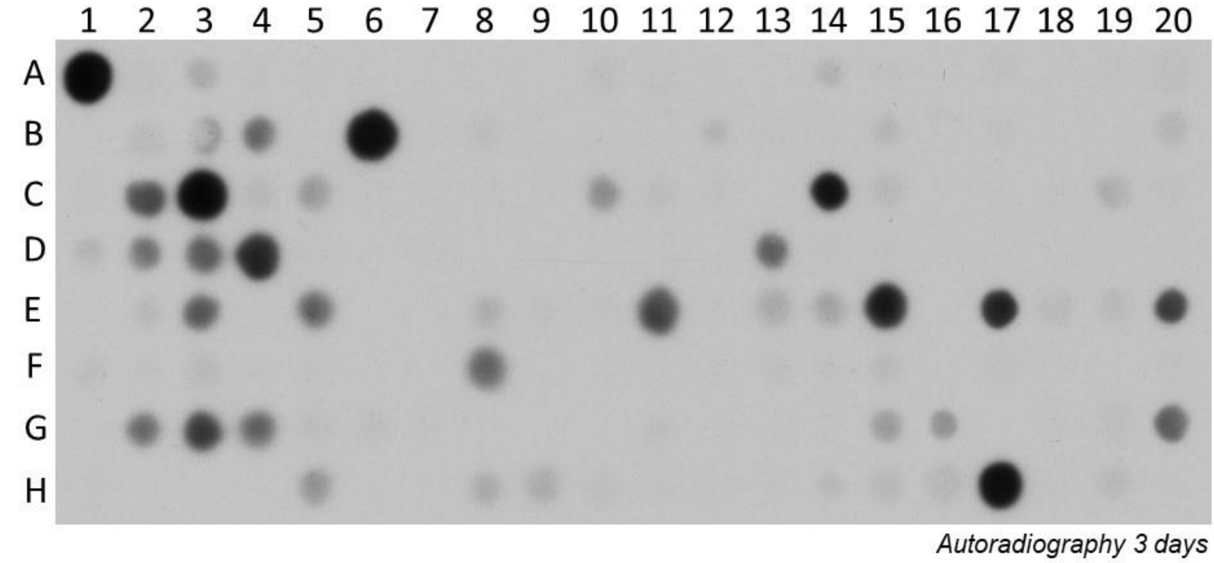


**Supplemental Figure 3. Search for additional HEMK2 peptide substrate candidates.** 152 putative substrate proteins were selected for methylation analysis and corresponding 15 aa long peptides with the predicted target-K in the center were synthesized on a SPOT array and methylated by 1.6 µM HEMK2/TRMT112 for one hour. As positive controls ERF1-Q185K (Spot A1/H17) and H4K12 (Spot A3/H19), as well as their corresponding K-to-A mutants (Spot A2/H18 and A4/H20) were included (Supplemental Table 2).

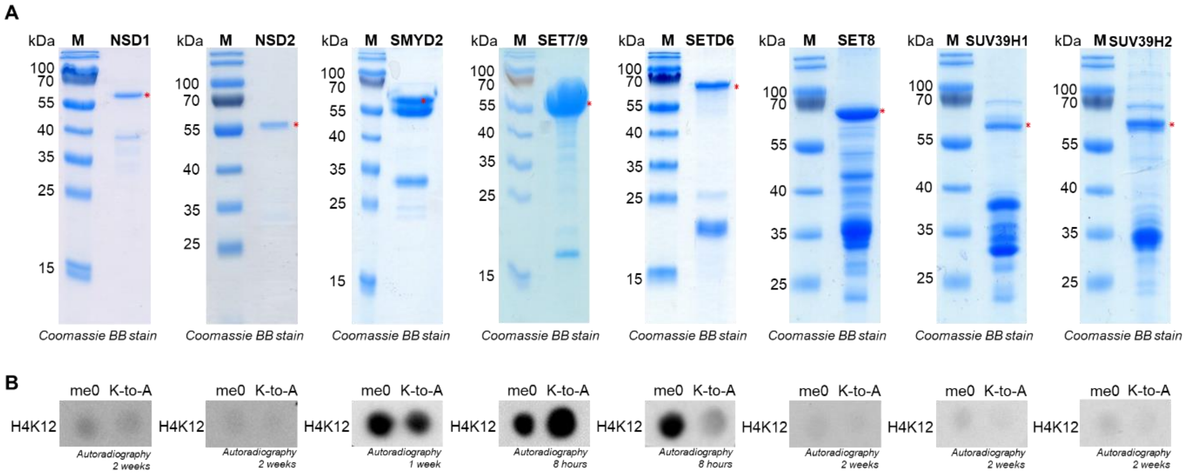


**Supplemental Figure 4. Purification and activity analysis of different PKMTs on the H4K12 peptide. A)** Coomassie stained SDS gel of purified NSD1, NSD2, SMYD2, SETD7/9, SETD6, SET8, SUV39H1 and SUV39H2 PKMTs. The bands at expected size are marked with red asterisks. **B)** SPOT peptide arrays were synthesized with H4K12 peptides and H4K12A as negative control. The peptide arrays were methylated with each enzyme in presence of radioactively labelled [methyl-$^{3}$H]-AdoMet for one hour. Methyl group transfer was detected by autoradiography after indicated film exposition times.

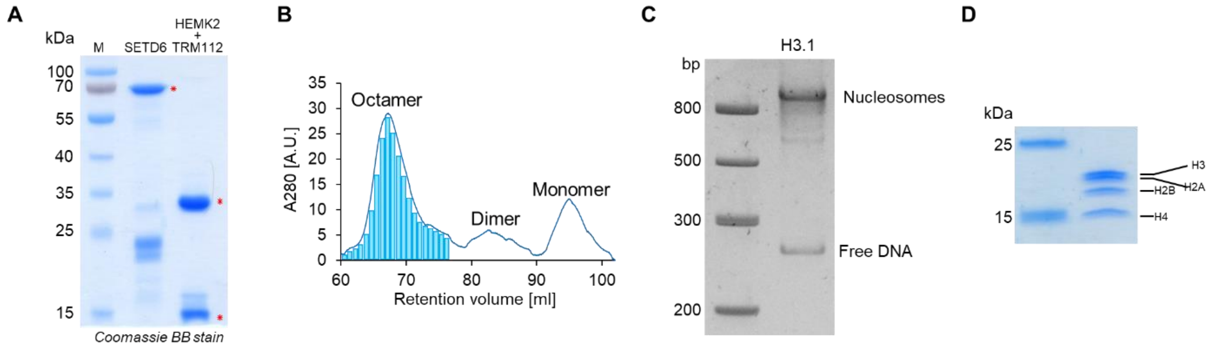


**Supplemental Figure 5. Loading controls and experiments for recombinant H3.1 nucleosome formation and methlyation. A)** Equal amounts of SETD6 and HEMK2/TRMT112 were loaded on an SDS-PAGE and used for methylation of GST-tagged H4 or GST-tagged H4K12Q protein. The bands at expected sizes are marked with red asterisks. **B)** Chromatogram of the histone octamer purification by size exclusion chromatography. Blue bars indicate the fractions that were collected and used for nucleosome reconstitution. **C)** Electrophoretic mobility gel shift assay of reconstituted nucleosomes showing the incorporation of DNA into the nucleosomes. **D)** Coomassie stained Tricine SDS-PAGE of the histone proteins in reconstituted nucleosomes showing the equal contents of all 4 histone proteins.

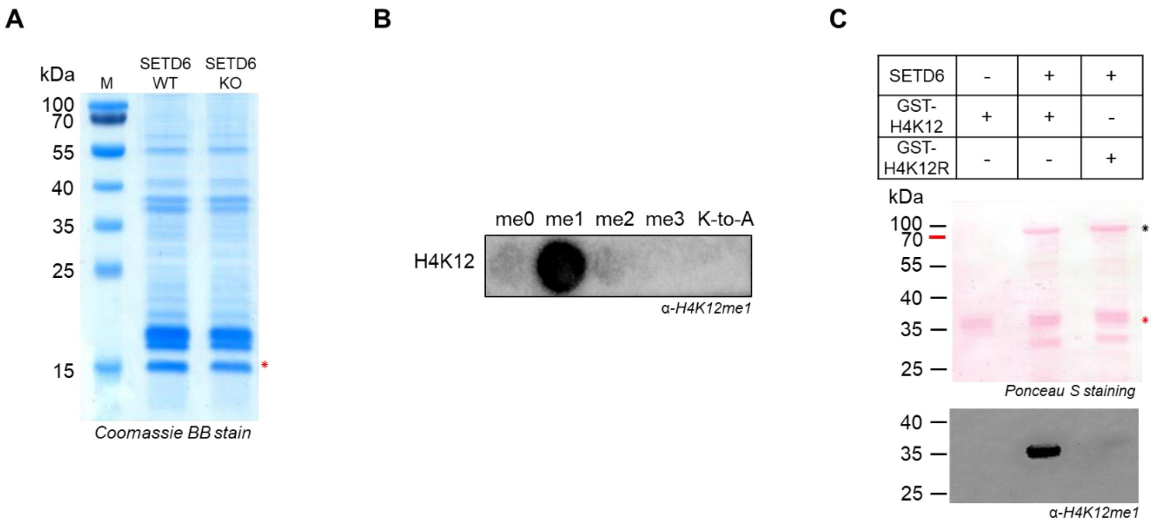


**Supplemental Figure 6. Loading controls and H4K12me1 antibody validation. A)** Histones were isolated from SETD6 WT and KO DU145 cells and separated by SDS-PAGE. Histone H4 is marked with a red asterisk. **B)** The H4K12me1 antibody was validated by peptide array binding. The peptide array consists of four spots corresponding to four different K12 methylation states of the H4K12 peptide (aa 6-19) (unmodified K12, H4K12me1, H4K12me2, H4K12me3) and as negative control H4K12A. The signal was detected by chemiluminescence after the addition of Pierce ECL Western Blotting substrate. **C)** GST-tagged H4 protein or the GST-tagged H4K12R protein (1.5 µM each) was incubated with SETD6 (2.5 µM) for 3 hours in the presence of 10 mM unlabeled AdoMet. Equal loading of SETD6 is indicated by a black asterisk, while equal loading of GST-tagged H4 proteins is marked with red asterisk in the Ponceau S staining. The methylation signal was detected by western blot with the H4K12me1-specific antibody.

## Supplemental Tables

### Supplemental Table 1: Sequences of the peptide spots shown in Figure 1B.

| | Swiss Prot No | ID | Protein name | Sequence | Target position |
|---|---|---|---|---|---|
| A1 | P62805 | H4 | Histone H4 | A G G K G L G K G G A K R H R | 12 |
| A2 | P62805 | H4K12Q | Histone H4 | A G G K G L G Q G G A K R H R | 12 |
| B1 | P62495 | ERF1 | Eukaryotic peptide chain release factor subunit 1 | K H G R G G Q S A L R F A R L | 185 |
| B2 | P62495 | ERF1-Q185K | Eukaryotic peptide chain release factor subunit 1 | K H G R G G K S A L R F A R L | 185 |
| C1 | Q8TDI0 | CHD5 | Chromodomain-helicase-DNA-binding protein 5 | E E R P E G Q S G R R Q S R R | 1390 |
| C2 | Q8TDI0 | CHD5-Q1390K | Chromodomain-helicase-DNA-binding protein 5 | E E R P E G K S G R R Q S R R | 1390 |
| D1 | Q86Y26 | NUT | NUT family member 1 | H H A S G G Q G S Q R A S H L | 1046 |
| D2 | Q86Y26 | NUT-Q1046K | NUT family member 1 | H H A S G G K G S Q R A S H L | 1046 |

**Supplemental Table 2: Sequences of the peptide spots shown in Supplemental Figure 3.**

| | Swiss Prot No | ID | Protein name | Sequence | Target position |
|---|---|---|---|---|---|
| A1 | Q9VPH7 | ERF1-Q185K | Eukaryotic peptide chain release factor subunit 1 | K H G R G G K S A L R F A R L R | 185 |
| A2 | Q9VPH7 | ERF1-Q185A | Eukaryotic peptide chain release factor subunit 1 | K H G R G G A S A L R F A R L R | 185 |
| A3 | P62805 | H4K12 | H4K12 | A G G K G L G K G G A K R H R A | 12 |
| A4 | P62805 | H4K12A | H4K12A | A G G K G L G A G G A K R H R A | 12 |
| A5 | Q86XS5 | ANGL5 | Angiopoietin-related protein 5 | V S Y T R S T K K L L R N M M D | 95 |
| A6 | P19801 | AOC1 | Amiloride-sensitive amine oxidase [copper-containing] | V H S F L W S K K E L R L Q P S | 51 |
| A7 | P25054 | APC | Adenomatous polyposis coli protein | W R A D V N S K K T L R E V G S | 560 |
| A8 | O95996 | APCL | Adenomatous polyposis coli protein 2 | W R A D I N S K K V L R E A G S | 525 |
| A9 | P98196 | AT11A | Phospholipid-transporting ATPase IH | D V L F E L S K T V L R H S G S | 732 |
| A10 | Q9Y5Z6 | B3GT1 | Beta-1,3-galactosyltransferase 1 | M H R I W N D M S S K K H L R C | 321 |
| A11 | Q00973 | B4GN1 | Beta-1,4 N-acetylgalactosaminyltransferase 1 | A L V T I A T K T F L R Y D R L | 284 |
| A12 | Q8NHY0 | B4GN2 | Beta-1,4 N-acetylgalactosaminyltransferase 2 | N L V T I A T K T F L R P H K L | 328 |
| A13 | Q8TBE0 | BAHD1 | Bromo adjacent homology domain-containing 1 protein | A K P P S G S K S G L R T G S S | 548 |
| A14 | A6QL63 | BTBDB | Ankyrin repeat and BTB/POZ domain-containing protein BTBD11 | L C A S R N S K A K L R A L R E | 789 |
| A15 | Q68D86 | C102B | Coiled-coil domain-containing protein 102B | G S G N G E T K T G L R L K A I | 238 |
| A16 | O60729 | CC14B | Dual specificity protein phosphatase CDC14B | S S V K S L S I S R T K T V L R | 486 |
| A17 | Q9NVL8 | CC198 | Uncharacterized protein CCDC198 | G L S H S K T H L R V I K V A P | 7 |
| A18 | Q6P9F0 | CCD62 | Coiled-coil domain-containing protein 62 | S H S L G S S K S A L R E D E T | 584 |
| A19 | Q9NVE4 | CCD87 | Coiled-coil domain-containing protein 87 | M T I K Y S S K A R L R Q L P S | 708 |
| A20 | Q86Y33 | CD20B | Cell division cycle protein 20 homolog B | Q L W D V V T K K R L R N M L G | 305 |
| B1 | Q5VT06 | CE350 | Centrosome-associated protein 350 | W D A L S Q T K A A L R H I E N | 38 |
| B2 | Q9C0B2 | CFA74 | Cilia- and flagella-associated protein 74 | V L V H T R S K A A L R L K F E | 831 |
| B3 | Q9Y4C5 | CHST2 | Carbohydrate sulfotransferase 2 | E E V K D L S K T L L R K P R L | 522 |
| B4 | Q8N3C7 | CLIP4 | CAP-Gly domain-containing linker protein 4 | R N A F S K S K A A L R R S W S | 601 |
| B5 | Q9HAW4 | CLSPN | Claspin | P M V I Q E S K S L L R N P F E | 1232 |
| B6 | G9CGD6 | CNIPF | CNK3/IPCEF1 fusion protein | G D R R P S T K K E L R K S F V | 797 |
| B7 | Q9BT09 | CNPY3 | Protein canopy homolog 3 | A S G V K Y T K S D L R L I E V | 91 |
| B8 | O94779 | CNTN5 | Contactin-5 | V N G Y I P S K A R L R K S Q A | 344 |
| B9 | P08684 | CP3A4 | Cytochrome P450 3A4 | D P F V E N T K K L L R F D F L | 208 |
| B10 | P24462 | CP3A7 | Cytochrome P450 3A7 | D P F V E N T K K L L R F N P L | 208 |
| B11 | Q92523 | CPT1B | Carnitine O-palmitoyltransferase 1, muscle isoform | M M E G S H T K A D L R D L F Q | 626 |
| B12 | Q68DL7 | CR063 | Uncharacterized protein C18orf63 | K P P N L T T K K M L R A S L T | 338 |
| B13 | Q9Y4K1 | CRBG1 | Beta/gamma crystallin domain-containing protein 1 | L M Q N L D T K S K L R P K R A | 776 |
| B14 | Q96PZ7 | CSMD1 | CUB and sushi domain-containing protein 1 | L G D D F K T K S L L R F S C E | 2938 |
| B15 | Q9UBT7 | CTNL1 | Alpha-catulin | M M L L T A S K T C L R H P N C | 243 |
| B16 | Q8WZ74 | CTTB2 | Cortactin-binding protein 2 | F D V D T L S K S E L R M L L S | 38 |
| B17 | Q9UKL4 | CXD2 | Gap junction delta-2 protein | S G L R T A S K S K L R R Q E G | 186 |
| B18 | Q7L576 | CYFP1 | Cytoplasmic FMR1-interacting protein 1 | I A D K S G S K K T L R S S L E | 577 |
| B19 | Q96F07 | CYFP2 | Cytoplasmic FMR1-interacting protein 2 | I A D K S G S K K T L R S S L D | 916 |
| B20 | Q9P219 | DAPLE | Protein Daple | A V E L A D T K A R L R R V R Q | 260 |
| C1 | Q008S8 | ECT2L | Epithelial cell-transforming sequence 2 oncogene-like | A I F L R C T K S Q L R F V Q D | 56 |
| C2 | Q5THR3 | EFCB6 | EF-hand calcium-binding domain-containing protein 6 | G Q N L T V S K S E L R R I I T | 373 |
| C3 | Q9UI10 | EI2BD | Translation initiation factor eIF-2B subunit delta | K V P A G R S K A E L R A E R R | 97 |
| C4 | Q5T0W9 | FA83B | Protein FAM83B | P T L E H T T K S F L R N W R I | 489 |
| C5 | Q9UKT4 | FBX5 | F-box only protein 5 | G P L P G T K K S K K N L R R L | 438 |

| | | | | | |
|---|---|---|---|---|---|
| C6 | Q9Y2I7 | FYV1 | 1-phosphatidylinositol 3-phosphate 5-kinase | L Y I R S H S K A V L R T S I H | 1995 |
| C7 | Q9UQC2 | GAB2 | GRB2-associated-binding protein 2 | Y Y K N D H S K K P L R I I N L | 55 |
| C8 | Q2WGN9 | GAB4 | GRB2-associated-binding protein 4 | Y Y K N D G S K K P L R T I N L | 87 |
| C9 | P48167 | GLRB | Glycine receptor subunit beta | C K K V C T S K S D L R S N D F | 412 |
| C10 | P49863 | GRAK | Granzyme K | K M L H I R S K T S L R S G T K | 140 |
| C11 | Q9H116 | GZF1 | GDNF-inducible zinc finger protein 1 | C G K G L S S K T A L R L H E R | 419 |
| C12 | Q4G0G2 | H1AS1 | Putative uncharacterized protein H1-10-AS1 | T S P F A M S K S S L R P P K K | 39 |
| C13 | Q7Z2G1 | H2BWT | Histone H2B type W-T | A E S E G T K A V L R T S L Y A | 160 |
| C14 | Q8WWN9 | ICEF1 | Interactor protein for cytohesin exchange factors 1 | G D R R P S T K K E L R K S F V | 336 |
| C15 | Q13099 | IFT88 | Intraflagellar transport protein 88 homolog | V R A A G F T K A A L R G S A F | 105 |
| C16 | O95259 | KCNH1 | Potassium voltage-gated channel subfamily H member 1 | S C D S G I T K S D L R L D N V | 886 |
| C17 | Q8NCM2 | KCNH5 | Potassium voltage-gated channel subfamily H member 5 | S C D S G I T K S D L R L D K A | 870 |
| C18 | Q7L273 | KCTD9 | BTB/POZ domain-containing protein KCTD9 | F L L A T P T K S E L R C Q G L | 215 |
| C19 | Q8IZU9 | KIRR3 | Kin of IRRE-like protein 3 | I N G A T Y S K T L L R D G K R | 197 |
| C20 | Q92615 | LAR4B | La-related protein 4B | P R S R N P S K S H L R H A I P | 415 |
| D1 | Q13136 | LIPA1 | Liprin-alpha-1 | R M L D H L T K K D L R G Q L K | 9998 |
| D2 | O75334 | LIPA2 | Liprin-alpha-2 | R M L D H L T K K D L R V H L K | 1055 |
| D3 | O75335 | LIPA4 | Liprin-alpha-4 | R M L D H L T K K D L R V H L K | 980 |
| D4 | Q8N8X9 | MB213 | Protein mab-21-like 3 | K D W Q V F S K A F L R L V R K | 306 |
| D5 | Q16539 | MK14 | Mitogen-activated protein kinase 14 | V C A A F D T K T G L R V A V K | 45 |
| D6 | O00566 | MPP10 | U3 small nucleolar ribonucleoprotein protein MPP10 | E R A E N S S K S D L R K S P V | 157 |
| D7 | A7E2Y1 | MYH7B | Myosin-7B | D R V S A L T K A K L R L E Q Q | 1071 |
| D8 | B2RTY4 | MYO9A | Unconventional myosin-IXa | H Y L N Q I T K K P L R Q S W D | 876 |
| D9 | Q6IA69 | NADE | Glutamine-dependent NAD(+) synthetase | N P I G G I S K T D L R A F V Q | 544 |
| D10 | Q5VST9 | OBSCN | Obscurin | S M G V S S T K A E L R V D L T | 5212 |
| D11 | Q8WVF1 | OSCP1 | Protein OSCP1 | K P Q E L Y S K K A L R T V Y E | 74 |
| D12 | Q7RTW8 | OTOAN | Otoancorin | T I A A G L T K A E L R M L D K | 1037 |
| D13 | Q8TDX9 | PK1L1 | Polycystic kidney disease protein 1-like 1 | R W A H P P S K A Q L R G T R Q | 2264 |
| D14 | Q15149 | PLEC | Plectin | A K L L N S S K A R L R S L E S | 746 |
| D15 | Q96I23 | PREY | Protein preY, mitochondrial | F L V C P L S K K P L R Y E A S | 64 |
| D16 | P23471 | PTPRZ | Receptor-type tyrosine-protein phosphatase zeta | A S L N D G S K T V L R S P H M | 543 |
| D17 | O75943 | RAD17 | Cell cycle checkpoint protein RAD17 | M S K T F L R P K V S S T K V T | 9 |
| D18 | P0DJH9 | RD3L | Protein RD3-like | P G S D I V T K T L L R E L K W | 28 |
| D19 | P84095 | RHOG | Rho-related GTP-binding protein RhoG | P I L L V G T K K D L R A Q P D | 116 |
| D20 | P13489 | RINI | Ribonuclease inhibitor | L C G I V A S K A S L R E L A L | 227 |
| E1 | Q9Y265 | RUVB1 | RuvB-like 1 | H L G E I G T K T T L R Y S V Q | 168 |
| E2 | Q08357 | S20A2 | Sodium-dependent phosphate transporter 2 | G D T V S Y S K K R L R Y D S Y | 425 |
| E3 | Q96K37 | S35E1 | Solute carrier family 35 member E1 | S L Q N I F S K K V L R D S R I | 208 |
| E4 | Q96HI0 | SENP5 | Sentrin-specific protease 5 | K H F I S S S K T L L R L Q A E | 109 |
| E5 | Q13435 | SF3B2 | Splicing factor 3B subunit 2 | P E A P K L S K K K L R R M N R | 451 |
| E6 | Q96B97 | SH3K1 | SH3 domain-containing kinase-binding protein 1 | S Q D E Q L S K S S L R E T T G | 172 |
| E7 | Q11203 | SIAT6 | CMP-N-acetylneuraminate-beta-1,4-galactoside alpha-2,3-sialyltransferase | F E K D V G S K T T L R I T Y P | 202 |
| E8 | Q92543 | SNX19 | Sorting nexin-19 | T E G K K A S K S R L R F S S S | 721 |
| E9 | Q9BQ16 | TICN3 | Testican-3 | G S Q N K K T K T L L R P E R S | 231 |
| E10 | Q8IWZ5 | TRI42 | Tripartite motif-containing protein 42 | R S I H T S S K T A L R T G S S | 116 |
| E11 | P0C672 | TSN19 | Tetraspanin-19 | Q V P C S C T K S T L R K W F C | 180 |
| E12 | Q96PF2 | TSSK2 | Testis-specific serine/threonine-protein kinase 2 | A E C K L D T K T G L R P D H R | 302 |
| E13 | Q96AY4 | TTC28 | Tetratricopeptide repeat protein 28 | R S L S V Q S K S H L R K N P P | 1466 |
| E14 | Q9NV66 | TYW1 | S-adenosyl-L-methionine-dependent tRNA 4-demethylwyosine synthase TYW1 | V K L C R W T K S M L R G R G G | 385 |
| E15 | Q9UPU5 | UBP24 | Ubiquitin carboxyl-terminal hydrolase 24 | F Q T Y L R T K K K L R V D T E | 2181 |

| | | | | | |
|---|---|---|---|---|---|
| E16 | P40818 | UBP8 | Ubiquitin carboxyl-terminal hydrolase 8 | A L F K W E S K T V L R N E P L | 287 |
| E17 | Q5T4S7 | UBR4 | E3 ubiquitin-protein ligase UBR4 | G N G K A P S K S E L R H L Y L | 4089 |
| E18 | Q96PU4 | UHRF2 | E3 ubiquitin-protein ligase UHRF2 | L K T I S R T K K E L R V K I F | 278 |
| E19 | Q8NB66 | UN13C | Protein unc-13 homolog C | H L G H M G S K A S L R F L N V | 318 |
| E20 | Q8NBZ7 | UXS1 | UDP-glucuronic acid decarboxylase 1 | M V S K A L L R L V S A V N R R | 4 |
| F1 | Q5THJ4 | VP13D | Vacuolar protein sorting-associated protein 13D | L L K R N C S K K P L R S R H S | 254 |
| F2 | A6NE52 | WDR97 | WD repeat-containing protein 97 | Q G P D L D S K A G L R T C C H | 1340 |
| F3 | Q9NPA5 | ZF64A | Zinc finger protein 64 | C D F L G D S K A T L R K H S R | 483 |
| F4 | Q9NPA5 | ZF64A | Zinc finger protein 64 | C S Y S C S S K A A L R I H E R | 507 |
| F5 | Q9NRM2 | ZN277 | Zinc finger protein 277 | C H V K F K S K A D L R T H M E | 369 |
| F6 | Q8NAF0 | ZN579 | Zinc finger protein 579 | E V D S K A H L R G L G G L A S | 501 |
| F7 | P17036 | ZNF3 | Zinc finger protein 69 | L N G I G M S K S S L R V T T E | 171 |
| F8 | Q6ZN30 | BNC2 | Zinc finger protein basonuclin-2 | L T K T E H P K S S F R I H R M | 548 |
| F9 | O00481 | BT3A1 | Butyrophilin subfamily 3 member A1 | R D G I T A G K A A L R I H N V | 421 |
| F10 | P78410 | BT3A2 | Butyrophilin subfamily 3 member A2 | R D G I T A G K A A L R I H N V | 108 |
| F11 | O00478 | BT3A3 | Butyrophilin subfamily 3 member A3 | R D G I T A G K A A L R I H N V | 108 |
| F12 | Q86Z23 | C1QL4 | Complement C1q-like protein 4 | G E V G R R G K A G L R G P P G | 108 |
| F13 | Q96LW7 | CAR19 | Caspase recruitment domain-containing protein 19 | A E K F R N P K A S L R V R L C | 45 |
| F14 | Q96MT7 | CFA44 | Cilia- and flagella-associated protein 44 | V T S K S D G K K S L R S S K S | 22 |
| F15 | Q9Y678 | COPG1 | Coatomer subunit gamma-1 | Q L F C S S P K A A L R Y A A V | 300 |
| F16 | Q9Y2R4 | DDX52 | Probable ATP-dependent RNA helicase DDX52 | D K L F E D G K T G F R D Q L A | 328 |
| F17 | Q9NYP3 | DONS | Protein downstream neighbor of Son | S Q I P L L G K S S L R N V V L | 549 |
| F18 | Q5RHP9 | ERIC3 | Glutamate-rich protein 3 | R R K A E R P K T S L R K T D S | 1067 |
| F19 | Q8N531 | FBXL6 | F-box/LRR-repeat protein 6 | P S A A A K P K A G L R S E A A | 82 |
| F20 | P20930 | FILA | Filaggrin | Q S V S G H G K A G L R Q Q S H | 442 |
| G1 | P06865 | HEXA | Beta-hexosaminidase subunit alpha | K E L E L V T K A G F R A L L S | 409 |
| G2 | Q14722 | KCAB1 | Voltage-gated potassium channel subunit beta-1 | M P H R N L G K S G L R V S C L | 95 |
| G3 | Q13303 | KCAB2 | Voltage-gated potassium channel subunit beta-2 | Q F Y R N L G K S G L R V S C L | 44 |
| G4 | O43448 | KCAB3 | Voltage-gated potassium channel subunit beta-3 | M K Y R N L G K S G L R V S C L | 88 |
| G5 | Q6P2M8 | KCC1B | Calcium/calmodulin-dependent protein kinase type 1B | V A L K C I P K K A L R G K E A | 49 |
| G6 | Q38SD2 | LRRK1 | Leucine-rich repeat serine/threonine-protein kinase 1 | L P S S Y P G K T A L R V K W S | 253 |
| G7 | Q14767 | LTBP2 | Latent-transforming growth factor beta-binding protein 2 | G G H G H D P K S G F R I Y F C | 392 |
| G8 | Q14566 | MCM6 | DNA replication licensing factor MCM6 | I N Q E S A P K A S L R L G F S | 710 |
| G9 | P50281 | MMP14 | Matrix metalloproteinase-14 | K V E P G Y P K S A L R D W M G | 499 |
| G10 | Q5JR59 | MTUS2 | Microtubule-associated tumor suppressor candidate 2 | A A K S N L P K S G L R P P G Y | 828 |
| G11 | Q8NEY1 | NAV1 | Neuron navigator 1 | D R N T L P K K G L R Y Q L Q S | 945 |
| G12 | Q8IVL1 | NAV2 | Neuron navigator 2 | D R N T L P K K G L R Y T P T S | 1514 |
| G13 | Q8IVL0 | NAV3 | Neuron navigator 3 | D R N T L P K K G L R Y T P S S | 1424 |
| G14 | Q5C9Z4 | NOM1 | Nucleolar MIF4G domain-containing protein 1 | A D L A T K C L Q G K A S L R M | 850 |
| G15 | P0C604 | OR4A8 | Olfactory receptor 4A8 | L L G Y Q S G K T G F R C S K L | 306 |
| G16 | Q9H5I5 | PIEZ2 | Piezo-type mechanosensitive ion channel component 2 | Q A W I T D P K T A L R Q R H K | 1591 |
| G17 | Q9BZ72 | PITM2 | Membrane-associated phosphatidylinositol transfer protein 2 | W F R S I L P K A A L R V V E E | 79 |
| G18 | Q6XQN6 | PNCB | Nicotinate phosphoribosyltransferase | K Q T L P G S K A A F R L L G S | 421 |
| G19 | Q96FQ6 | S10AG | Protein S100-A16 | L V K N K I S K S S F R E M L Q | 35 |
| G20 | Q9H190 | SDCB2 | Syntenin-2 | L C K D E R G K T G L R L R K V | 117 |
| H1 | Q9UHD8 | 40057 | Septin-9 | S L S Q R S P K A S L R R V E L | 86 |
| H2 | Q8ND04 | SMG8 | Protein SMG8 | C V V G I F G K T A L R L N S E | 55 |
| H3 | A2VDJ0 | T131L | Transmembrane protein 131-like | R V I P A M G K T S F R I I F L | 151 |
| H4 | Q0IIM8 | TBC8B | TBC1 domain family member 8B | V I I S I K G K T A F R F H E V | 336 |
| H5 | Q9C0I4 | THS7B | Thrombospondin type-1 domain-containing protein 7B | N C H D P Q G K K G F R T R Q R | 509 |

| | | | | | |
|---|---|---|---|---|---|
| H6 | Q8WZ42 | TITIN | Titin | T V T T E C S K T S F R V A N L | 20769 |
| H7 | P51580 | TPMT | Thiopurine S-methyltransferase | L D T F L K G K S G L R V F F P | 60 |
| H8 | Q9NV66 | TYW1 | S-adenosyl-L-methionine-dependent tRNA 4-demethylwyosine synthase TYW1 | V K L C R W T K S M L R G R G G | 385 |
| H9 | Q86UV5 | UBPB48 | Ubiquitin carboxyl-terminal hydrolase 48 | S D G F W V G K S S L R S W R Q | 596 |
| H10 | Q8NB66 | UN13C | Protein unc-13 homolog C | H L G H M G S K A S L R F L N V | 318 |
| H11 | A6NE52 | WDR97 | WD repeat-containing protein 97 | Q G P D L D S K A G L R T C C H | 1340 |
| H12 | Q9NTW7 | ZF64B | Zinc finger protein 64 | C S Y S C S S K A A L R I H E R | 356 |
| H13 | Q8NDP4 | ZN439 | Zinc finger protein 439 | W K S Q Q P K K A F R Y H P S L | 165 |
| H14 | Q8IYI8 | ZN440 | Zinc finger protein 440 | C K C Q Q P K K A F R Y R P S F | 151 |
| H15 | Q9Y4E5 | ZN459 | E3 SUMO-protein ligase ZNF451 | G T I N C G T K S S F R R G G H | 153 |
| H16 | Q9UC07 | ZNF69 | Zinc finger protein 69 | C K C Q Q P K K A F R Y H P S F | 171 |
| H17 | Q9VPH7 | ERF1-Q185K | Eukaryotic peptide chain release factor subunit 1 | K H G R G G K S A L R F A R L R | 185 |
| H18 | Q9VPH7 | ERF1-Q185A | Eukaryotic peptide chain release factor subunit 1 | K H G R G G A S A L R F A R L R | 185 |
| H19 | P62805 | H4K12 | H4K12 | A G G K G L G K G G A K R H R A | 12 |
| H20 | P62805 | H4K12A | H4K12A | A G G K G L G A G G A K R H R A | 12 |

## Supplemental Table 3: Sequences of the peptide spots shown in Figure 3A.

| | Swiss Prot No | ID | Protein name | Sequence | Target position |
|---|---|---|---|---|---|
| A1 | P62495 | ERF1-Q185K | Eukaryotic peptide chain release factor subunit 1 | K H G R G G K S A L R F A R L R | 185 |
| A2 | P62495 | ERF1-Q185A | Eukaryotic peptide chain release factor subunit 1 | K H G R G G A S A L R F A R L R | 185 |
| A3 | P62805 | H4 | Histone H4 | A G G K G L G K G G A K R H R A | 12 |
| A4 | P62805 | H4K12A | Histone H4 | A G G K G L G A G G A K R H R A | 12 |
| A5 | G9CGD6 | CNIPF | CNK3/IPCEF1 fusion protein | G D R R P S T K K E L R K S F V | 797 |
| A6 | G9CGD6 | CNIPF-K797A | CNK3/IPCEF1 fusion protein | G D R R P S T A K E L R K S F V | 797 |
| A7 | Q9UI10 | EI2BD | Translation initiation factor eIF-2B subunit delta | K V P A G R S K A E L R A E R R | 97 |
| A8 | Q9UI10 | EI2BD-K97A | Translation initiation factor eIF-2B subunit delta | K V P A G R S A A E L R A E R R | 97 |
| B1 | Q8WWN9 | ICEF1 | Interactor protein for cytohesin exchange factors 1 | G D R R P S T K K E L R K S F V | 336 |
| B2 | Q8WWN9 | ICEF1-K336A | Interactor protein for cytohesin exchange factors 1 | G D R R P S T A K E L R K S F V | 336 |
| B3 | Q8N8X9 | MB213 | Protein mab-21-like 3 | K D W Q V F S K A F L R L V R K | 306 |
| B4 | Q8N8X9 | MB213-K306A | Protein mab-21-like 3 | K D W Q V F S A A F L R L V R K | 306 |
| B5 | P0C672 | TSN19 | Tetraspanin-19 | Q V P C S C T K S T L R K W F C | 180 |
| B6 | P0C672 | TSN19-K180A | Tetraspanin-19 | Q V P C S C T A S T L R K W F C | 180 |
| B7 | Q9UPU5 | UBP24 | Ubiquitin carboxyl-terminal hydrolase 24 | F Q T Y L R T K K K L R V D T E | 2181 |
| B8 | Q9UPU5 | UBP24-K2181A | Ubiquitin carboxyl-terminal hydrolase 24 | F Q T Y L R T A K K L R V D T E | 2181 |
| C1 | Q5T4S7 | UBR4 | E3 ubiquitin-protein ligase UBR4 | G N G K A P S K S E L R H L Y L | 4089 |
| C2 | Q5T4S7 | UBR4-K4089A | E3 ubiquitin-protein ligase UBR4 | G N G K A P S A S E L R H L Y L | 4089 |
| C3 | Q8NBZ7 | UXS1 | UDP-glucuronic acid decarboxylase 1 | M V S K A L L R L V S A V N R R | 4 |
| C4 | Q8NBZ7 | UXS1-K4A | UDP-glucuronic acid decarboxylase 1 | M V S A A L L R L V S A V N R R | 4 |
| C5 | Q13303 | KCAB2 | Voltage-gated potassium channel subunit beta-2 | Q F Y R N L G K S G L R V S C L | 44 |
| C6 | Q13303 | KCAB2-K44A | Voltage-gated potassium channel subunit beta-2 | Q F Y R N L G A S G L R V S C L | 44 |
| C7 | P62495 | ERF1-Q185K | Eukaryotic peptide chain release factor subunit 1 | K H G R G G K S A L R F A R L R | 185 |
| C8 | P62495 | ERF1-Q185A | Eukaryotic peptide chain release factor subunit 1 | K H G R G G A S A L R F A R L R | 185 |

**Supplemental Table 4: Sequences of the peptide spots shown in Figure 5A.**

| Swiss Prot No | ID | Protein name | Sequence |
|---|---|---|---|
| P62805 | H4K12 | Histone H4 | A G G K G L G K G G A K R H R |
| P62805 | H4K12me1 | Histone H4 | A G G K G L G Kme1 G G A K R H R |
| P62805 | H4K12me2 | Histone H4 | A G G K G L G Kme2 G G A K R H R |
| P62805 | H4K12me3 | Histone H4 | A G G K G L G Kme3 G G A K R H R |
| P62805 | H4K12A | Histone H4 | A G G K G L G A G G A K R H R |

**Supplemental Table 5: Sequences of the peptide spots shown in Figure 5B and C.**

| | Swiss Prot No | ID | Protein name | Sequence |
|---|---|---|---|---|
| A1 | P62495 | ERF1 | Eukaryotic peptide chain release factor subunit 1 | K H G R G G K S A L R F A R L |
| A2 | P62495 | ERF1-Q185A | Eukaryotic peptide chain release factor subunit 1 | K H G R G G A S A L R F A R L |
| A3 | Q04206 | RELA | Transcription factor p65 | K R T Y E T F K S I M K K S P |
| A4 | Q04206 | RELA-K310A | Transcription factor p65 | K R T Y E T F A S I M K K S P |
| B1 | P62805 | H4K12 | Histone H4 | A G G K G L G K G G A K R H R |
| B2 | P62805 | H4K8A | Histone H4 | A G G A G L G K G G A K R H R |
| B3 | P62805 | H4K12A | Histone H4 | A G G K G L G A G G A K R H R |
| B4 | P62805 | H4K16A | Histone H4 | A G G K G L G K G G A A R H R |
| C1 | P62805 | H4K8A/K12A | Histone H4 | A G G A G L G A G G A K R H R |
| C2 | P62805 | H4K8A/K16A | Histone H4 | A G G A G L G K G G A A R H R |
| C3 | P62805 | H4K12A/K16A | Histone H4 | A G G K G L G A G G A A R H R |
| C4 | P62805 | H4K8A/K12A/K16A | Histone H4 | A G G A G L G A G G A A R H R |


1. Kusevic D, Kudithipudi S, Jeltsch A. 2016. Substrate specificity of the hemk2 protein glutamine methyltransferase and identification of novel substrates. The Journal of biological chemistry. 291(12):6124-6133.
2. Metzger E, Wang S, Urban S, Willmann D, Schmidt A, Offermann A, Allen A, Sum M, Obier N, Cottard F et al. 2019. Kmt9 monomethylates histone h4 lysine 12 and controls proliferation of prostate cancer cells. Nature structural & molecular biology. 26(5):361-371.
3. Graille M, Heurgue-Hamard V, Champ S, Mora L, Scrima N, Ulryck N, van Tilbeurgh H, Buckingham RH. 2005. Molecular basis for bacterial class i release factor methylation by prmc. Molecular cell. 20(6):917-927.
